# Effect of cryopreservation medium conditions on growth and isolation of gut anaerobes from human faecal samples

**DOI:** 10.1186/s40168-022-01267-2

**Published:** 2022-05-30

**Authors:** Anaïs Biclot, Geert R. B. Huys, Rodrigo Bacigalupe, Kevin D’hoe, Doris Vandeputte, Gwen Falony, Raul Y. Tito, Jeroen Raes

**Affiliations:** 1 Laboratory of Molecular Bacteriology, Department of Microbiology and Immunology, Rega Institute, KU Leuven, Leuven, Belgium; 2 Center for Microbiology, VIB, Leuven, Belgium; 3 Research Group of Microbiology, Department of Bioengineering Sciences, Vrije Universiteit Brussel, Brussels, Belgium; 4 Present address: Meinig School of Biomedical Engineering, Cornell, USA

## Abstract

**Background:**

Novel strategies for anaerobic bacterial isolations from human faecal samples and various initiatives to generate culture collections of gut-derived bacteria have instigated considerable interest for the development of novel microbiota-based treatments. Early in the process of building a culture collection, optimal faecal sample preservation is essential to safeguard the viability of the broadest taxonomic diversity range possible. In contrast to the much more established faecal storage conditions for meta-omics applications, the impact of stool sample preservation conditions on bacterial growth recovery and isolation remains largely unexplored. In this study, aliquoted faecal samples from eleven healthy human volunteers selected based on a range of physicochemical and microbiological gradients were cryopreserved at – 80 °C either without the addition of any medium (dry condition) or in different Cary-Blair medium conditions with or without a cryoprotectant, i.e. 20% (v/v) glycerol or 5% (v/v) DMSO. Faecal aliquots were subjected to bulk 16S rRNA gene sequencing as well as dilution plating on modified Gifu Anaerobic Medium after preservation for culturable fraction profiling and generation of bacterial culture collections.

**Results:**

Analyses of compositional variation showed that cryopreservation medium conditions affected quantitative recovery but not the overall community composition of cultured fractions. Post-preservation sample dilution and richness of the uncultured source samples were the major drivers of the cultured fraction richness at genus level. However, preservation conditions differentially affected recovery of specific genera. Presence-absence analysis indicated that twenty-two of the 45 most abundant common genera (>0.01% abundance, dilution 10^−4^) were recovered in cultured fractions from all preservation conditions, while nine genera were only detected in fractions from a single preservation condition. Overall, the highest number of common genera (i.e. 35/45) in cultured fractions were recovered from sample aliquots preserved without medium and in the presence of Cary-Blair medium containing 5% (v/v) DMSO. Also, in the culture collection generated from the cultured fractions, these two preservation conditions yielded the highest species richness (72 and 66, respectively).

**Conclusion:**

Our results demonstrate that preservation methods partly determine richness and taxonomic diversity of gut anaerobes recovered from faecal samples. Complementing the current standard practice of cryopreserving stool samples in dry conditions with other preservation conditions, such as Cary-Blair medium with DMSO, could increase the species diversity of gut-associated culture collections.

Video abstract

**Supplementary Information:**

The online version contains supplementary material available at 10.1186/s40168-022-01267-2.

## Background

While metagenomic profiling has become the standard approach to study gut microbial ecosystems, awareness is growing that parallel efforts in cultivation-based approaches are indispensable to fully comprehend the functional capacities of its members and their interactions [1]. The availability of pure microbial cultures isolated from human gut samples is one of the key elements in the biological validation of host-microbiota associations inferred from metagenomics-based readouts [2]. In addition, building culture collections of gut anaerobes can also promote bioprospection of strains with novel functionalities and the description of novel species [3–5] and, ultimately, lead to the development of new biotherapeutics [6].

From sample collection to strain cataloguing, the generation of microbial culture collections can be a long and complex process. For gut-derived collections, safeguarding anaerobic conditions during faecal sampling and processing to minimize losses in viability and culturability of oxygen-sensitive organisms poses an additional challenge. Although immediate processing after sampling is highly preferred for microbial isolation purposes, this is not always possible due to complex logistics and sample sizes of most human cohort studies. Therefore, intermediate sample preservation is often a necessity during large-scale sampling campaigns, given that cultivation is a lengthy process and sample throughput is usually limited. Many different methods for long-time cryopreservation of faecal specimens have been described, all of which usually involve freezing at − 20°C or − 80°C with or without the addition of cryoprotectants (e.g. glycerol or dimethyl sulfoxide - DMSO) and/or protective buffers [7–9]. In the gut microbiome field, evaluation of these methods has mostly focused on the integrity of nucleic acids [10, 11] rather than on the recovery of culturable microbes. Yet, DNA-based profiling and microbial isolation are very different processes. When comparing sample preservation conditions, it is not only necessary to investigate how community composition and diversity are potentially impacted, but also to assess if different conditions ensure viability and would promote growth and isolation of taxonomically different strain sets.

To date, reports on the recovery of (living) faecal communities or specific commensal gut species are very limited. Alternatively, important insights can be obtained from studies on the post-preservation recovery of artificial microbial communities and fermented faecal samples. Kerckhof and colleagues reported that overall community compositions of faecal fermentation samples after preservation in DMSO (5% v/v), DMSO with trehalose and tryptic soy (5%, 1%, 0.3% v/v), or without protective agents were not impacted by any condition [12]. The authors pointed out that 15% of operational taxonomy units (OTUs) were not recovered in at least one condition; however, these OTUs were recovered better after preservation with cryoprotective agents. Similarly, Bircher and colleagues showed that butyrate producers recovered well from artificial microbiota cryopreserved in a medium containing glycerol and inulin (15%, 5% v/v) [13]. These studies reinforce the important role of cryoprotective media in maintaining community integrity and recovery of taxa in complex communities. Still, taxon-specific recovery differences are likely to occur under any given cryopreservation condition [12]. In another study Bircher and colleagues confirmed species-specific differences in the viability of obligately anaerobic gut isolates following cryopreservation at − 80°C in sucrose and inulin (5% v/v each), sucrose, inulin and glycerol (5%, 5% and 15% v/v), lyophilisation, or storage at 4°C. Overall, species best recovered from cryopreservation in the sucrose, inulin and glycerol combination, while lyophilisation was more detrimental [14].

While in previous studies different temperatures were tested on the recovery of communities and isolates, the impact of the medium was not. In the current study, we compared four preservation conditions that differed in the addition of Cary Blair (CB) medium and/or the choice of cryoprotectant added while keeping the storage temperature constant at − 80°C. CB is an isotonic nutrient-free transport medium for faecal samples characterized by a low oxidation-reduction potential and a high pH of 8.4 to protect bacterial cells from acidic shifts [15, 16]. We assessed to what extent these different cryopreservation medium conditions impacted microbial richness and culture recovery for a selection of faecal samples collected from 11 healthy human volunteers. In a parallel approach, samples were subjected to both culture-independent 16S rRNA gene community profiling as well as (after preservation) dilution plating for the purpose of culturable fraction profiling and isolation of pure cultures. Culture-based assessments were performed using modified Gifu Anaerobic Medium (mGAM), one of the most commonly used non-selective general media for intestinal anaerobes known to support the growth of most predominant gut species [3, 17, 18]. Comparison of our results across different conditions tested suggests that cryopreservation of faecal sample aliquots under multiple medium conditions guarantees a larger taxonomic spectrum of isolated species compared to preservation using one single condition, guiding future gut culturomics efforts.

## Methods

### Ethical compliance

All experimental protocols were approved by the Commissie Medische Ethiek, UZ KU Leuven. Study design complied with all relevant ethical regulations, aligning with the Declaration of Helsinki and in accordance with Belgian privacy laws. All participants gave their informed consent. Ethical approval of the study protocol was obtained (S59288).

### Sample collection and preservation

**Fig. 1:**
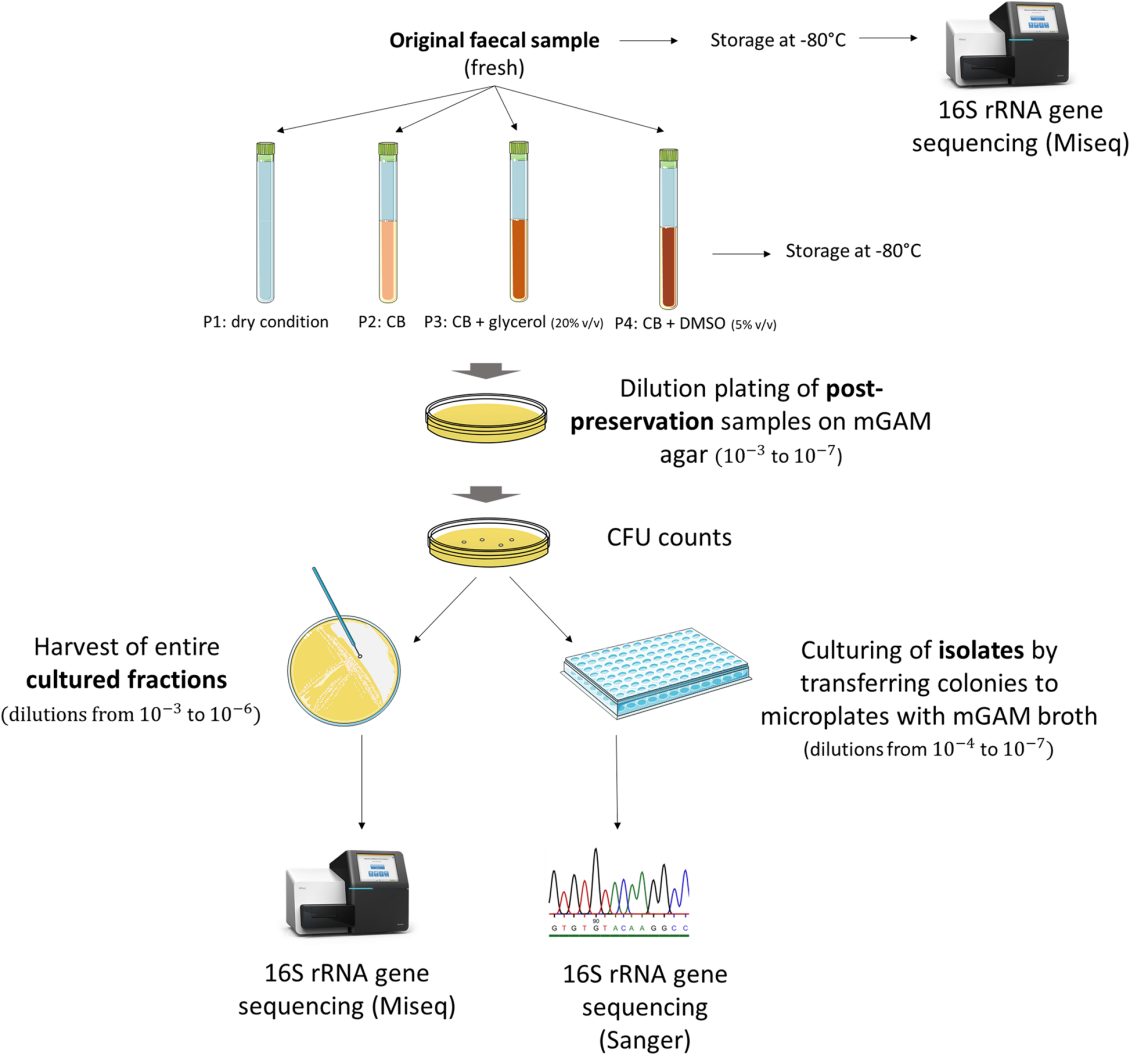
Experimental setup of the study. Aliquots of original fresh samples were used for community profiling and for comparison of post-preservation samples kept at − 80°C in four different preservation conditions with or without medium and/or cryoprotectants: dry (P1), CB (P2), CB + glycerol (20%) (P3), and CB + DMSO (5%) (P4). Following preservation, sample dilutions for each condition were plated on mGAM and incubated anaerobically. After 48h, CFU count was determined, and colonies were selected from countable plates for isolation in 96-well microplates and subsequent identification by Sanger 16S rRNA gene sequencing. From the same dilution series, plates with high colony numbers were fully harvested as cultured fractions and subjected to MiSeq community profiling

An initial cohort of 51 healthy donors from the KU Leuven community provided the faecal samples used for this study [19]. No inclusion or exclusion criteria were imposed. A limited set of anthropometric metadata was compiled at enrolment, including gender, age, height, and weight. Participants were asked to collect a maximal amount of faeces (single defecation) in a plastic receptacle with a lid and to deposit the sample in a labelled non-transparent zip-lock bag at the research facility immediately after defecation. All faecal samples were processed within an hour of the reported time of egestion. Upon collection, fresh faecal samples were homogenized and divided in a series of aliquots for physicochemical analyses, 16S rRNA gene community profiling and comparative analyses of four different conditions. The latter comparison required two aliquots with specific weights. First, 1 g of fresh faecal sample was stored dry in a cryotube without the addition of a preservation medium or cryoprotectant (preservation condition P1). In parallel, 3.75 g of fresh sample was resuspended in 15 mL of sterile Cary-Blair transport medium with red phenol indicator (Remel, Lenexa, USA). The resulting suspension was distributed over three different cryotubes each containing four mL of faecal suspension. The first tube was preserved without a cryoprotective agent (preservation condition P2), the second was supplemented with 1 mL sterile glycerol (Alfa Aesar, Thermofisher, Germany) to a final concentration of 20% v/v (preservation condition P3) and the third with 0.2 mL DMSO (Sigma Aldrich, USA) to a final concentration of 5% v/v (preservation condition P4; Fig. 1).

### Faecal samples selection

Out of the initial 51 faecal sample donors, 11 were selected along a gradient of physicochemical (i.e. faecal pH, water activity, and moisture content) and microbiological (i.e. microbial load) parameters (Supplementary Table S1). Water activity was measured in duplicate at 37°C using a resistive electrolytic hygrometer (Labmaster, Novasina, Lachen, Switzerland) after manual homogenization of the fresh faecal sample. Faecal pH of fresh samples was measured in triplicate after mechanical homogenization (5 min, 150 r.p.m; Stomacher 3500 [Seward Ltd., Worthing, UK]) using a FG2 pH meter coupled to an InLab Solids electrode (Mettler Toledo, Greifensee, Switzerland). Stool moisture content was determined in duplicate on 0.2 g of frozen faecal material (-80°C) as the percentage of mass loss after lyophilization. Microbial load of frozen cells was determined in triplicate using a C6 Accuri flow cytometer (BD Biosciences, New Jersey, USA) as described previously [19].

### Cultivation, colony counts and isolation

All post-preservation sample dilution, culturing and isolation steps were performed at 37°C under anaerobic conditions (10% H_2_, 10% CO_2_, 80% N_2_) in a Don Whitley A35 Anaerobic Workstation with HEPA filter (Don Whitley Scientific, Shipley, UK). All dilution and culture media were anaerobically preincubated overnight.

Per post-preservation sample, a dilution series (10^−1^ to 10^−7^) for each preservation condition was prepared in PBS and dilutions 10^−3^ to 10^−7^ were plated in triplicate on modified Gifu Anaerobic Medium (mGAM; Hyserve) agar and incubated for 5 days. Plates corresponding to countable dilutions (i.e. plate dilutions producing CFU in the range of 20–200, *n*=203) were first subjected to CFU counting and subsequently used for isolation. Colonies were selected (i.e. 48 colonies per countable dilution when possible, up to 95 per preservation condition) for isolation based on morphological differences (i.e. diameter, edge, shape and colour) and were transferred to a 96-well microplate in mGAM broth. Following incubation for 48h, 15% (v/v) glycerol was added and homogenized into each well after which the microwell plates were stored at − 80°C to use for identification by partial 16S rRNA gene sequencing. For all dilutions that yielded >100 colonies, one plate was kept aside for harvesting its entire biomass (from here on referred to as cultured fraction) which was resuspended in PBS. Following centrifugation (18,000×*g*, 2 min) and removal of supernatant, these fractions were stored at − 80°C for further 16S rRNA gene MiSeq community profiling. The entire experimental design is visualized in Fig. 1.

### Community profiling of faecal samples and cultured fractions

Before processing for 16S rRNA gene-based community profiling, cultured fraction pellets were resuspended in 4.5 mL PBS (Sigma Aldrich, USA) and split in 3 aliquots of 1.5 mL. Samples were centrifuged and supernatant was removed prior to storage at − 80°C in cryovials. Frozen faecal aliquots (between 150 and 200 mg) and cultured fractions were subjected to DNA extraction using the PowerMicrobiome DNA/RNA Isolation Kit (MO BIO Laboratories Inc., Carlsbad, USA) according to the manufacturer’s instructions as described previously [20]. The V4 hypervariable region of the 16S rRNA gene was amplified using forward primer 515F (5′ GTGYCAGCMGCCGCGGTAA 3′) and the reverse primer 806R (5′ GGACTACNVGGGTWTCTAAT 3′), modified with adapters and barcodes [20]. Sequencing was then performed using the Illumina MiSeq platform (MiSeq Reagent Kit v2, 500 cycles, 20% PhiX) according to the manufacturer’s specifications to generate paired-end reads of 250 bases in length in each direction. After demultiplexing with sdm as part of the LotuS [21] pipeline without allowing for mismatches, fastq sequences were pre-processed using DADA2 pipeline v1.14.1 [22]. The taxonomy was assigned using GTDB release 95 [23, 24]. For the comparison with FGFP [20] samples, taxonomy classification was also performed using the RDP classifier [25]. For relative microbiome analyses, each sample depth was rarefied to 10,000 reads.

### Identification of isolated colonies

From individual bacterial colony suspensions stored in 96-well microplates, 20 μL was transferred into a new microplate and washed with PBS to remove the excess medium. Plates were centrifuged for 10 min at 3,000 x g, after which the supernatant was removed. Following a second washing step, pellets were resuspended in 20 μL alkaline lysis solution containing 2.5 mL 10% SDS (Invitrogen, USA), 5.0 mL 1N NaOH (Merck, New Jersey, USA) and 92.5 mL sterile MilliQ per 100 mL. Samples were heated at 95°C for 15 min and immediately put on ice for 10 min. Finally, 180 μL of sterile MilliQ water was added and after gentle mixing, samples were centrifuged for 20 min at 3,000 x g and stored at − 20°C until further analysis. For identification based on partial 16S rRNA gene sequencing, 2 μL of each extract was used as a template for PCR with forward primer 27F (5′AGAGTTTGATCCTGGCTCAG 3′) and reverse primer 1492R (5′ ACGGCTACCTTGTTACGACTT 3′). Following agarose electrophoresis check, amplicons were sent for 16S rRNA gene Sanger sequencing to Eurofins (Germany). Sequences were taxonomically assigned by GTDB release 95 [23, 24].

### Statistical analyses

All statistical analyses and graphical representations were performed in R using the packages phyloseq [26] vegan [27], CoDaSeq [28] and ggplot2 [29]. Observed richness was calculated with the R package phyloseq. Microbiome variation between individuals was visualized by PCoA using Bray-Curtis distances, only samples with more than 10,000 reads and amplicon sequence variants (ASV) with a relative abundance higher than 0.001 across the dataset were included in the 16S rRNA data analysis. Correlations between continuous variables were analysed using non-parametric Spearman tests.

The contribution of metadata variables to the cultured fraction community variation was determined by distance-based redundancy analysis (dbRDA) on genus-level Aitchison distance (Bray-Curtis distances between samples after rarefication) with the capscale function in the vegan R package [27]. Correction for multiple testing (Benjamin–Hochberg procedure, FDR) was applied and significance was defined at FDR < 0.1. The cumulative contribution of metadata variables was determined by forward model selection on dbRDA with the ordiR2step function in vegan [27], with variables that showed a significant contribution to cultured fraction community variation in the previous step.

Alpha-diversity, number of observed genera, species and ASV (richness) of both the cultured fraction and faecal samples were determined using the vegan R package [27]. Associations between the richness and diversity of the cultured fractions and preservation conditions were assessed by fitting generalized linear models (GLMs). The model used multivariate analysis assessing the effect of preservation conditions, dilution factors and initial richness (RichnessF) from the faecal samples. The significance of was assessed by performing log-likelihood (*χ*^2^) tests.

$$\mathrm{Nullmodel}\ \mathrm{richness}=\mathrm{specnumber}\sim \mathrm{Dilution}+\mathrm{Preservation}+\mathrm{RichnessF}$$

with specnumber as the number of species found in the sample (richness).

## Results

### Faecal sample selection based on physicochemical and microbial parameters results in a wide spectrum of covered microbiota compositions

**Fig. 2:**
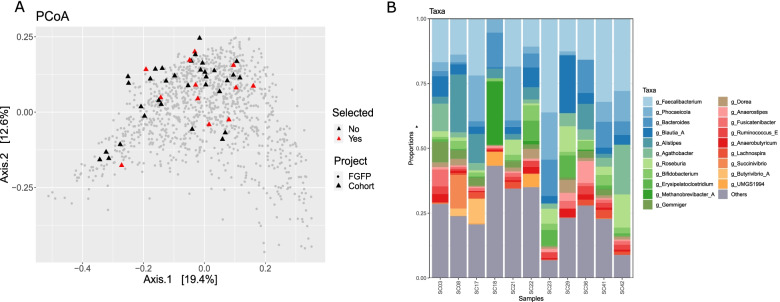
**A** Genus-level faecal microbiome community variation, represented by principal coordinates analysis (Bray–Curtis dissimilarity PCoA). Shown are samples from the original cohort (triangles, *n* = 51) further subdivided in a subset of 11 samples selected for this study (red triangles) and the non-selected samples (black triangles), as well as samples from the FGFP cohort [20] (dots, *n* = 1103). **B** Relative abundances of the top 20 genera from the 11 selected faecal samples derived from 16S rRNA gene community profiling

Faecal samples from eleven donors were selected from a larger cohort based on gradients [20] in faecal pH, water activity, moisture content, and microbial load (Supplementary Fig. S1). The four parameters did not show any significant cross-correlations (Spearman test, *p*>0.05, Supplementary Fig. S2). For each of the selected faecal samples, one aliquot was subjected to microbial community profiling in order to obtain a culture-independent baseline of the microbial diversity in each sample. Analysis of these profiles showed that the 11 faecal samples comprised a broad compositional diversity, as evidenced by their microbial community variation when compared to the larger Flemish Gut Flora Project [20] (FGFP) population (*n*=1103) (Fig. 2A) and by their diverse top 20 genera profiles (Fig. 2B). Thus, the sample selection process based on physicochemical and microbiological gradients resulted in a subset of faecal samples displaying a wide range of microbiota compositions.

### Quantitative recovery but not overall microbial community composition is impacted by preservation conditions in cultured fractions

**Fig. 3:**
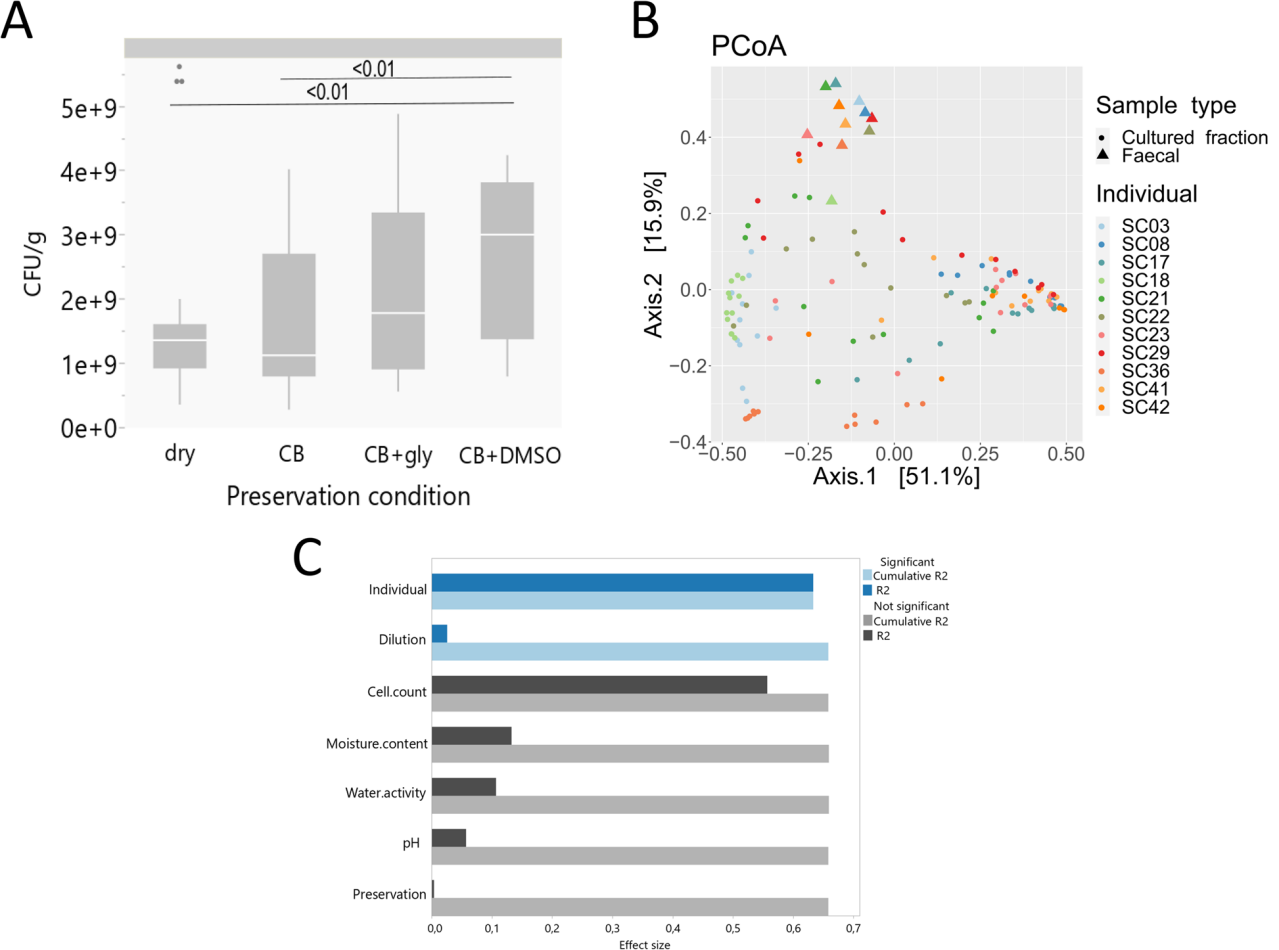
**A** CFU counts of cultured fractions obtained from dilution 10^−6^ in triplicate for each preservation condition including all individuals (*n*= 103), significant differences were observed between condition P4 and conditions P1 and P2, respectively (Kruskal–Wallis with post hoc Dunn test, *p <* 0.001; *p* < 0.01 for both). **B** Community variation of cultured fractions and original faecal samples at genus level visualized by principal coordinates analysis (genus level Bray-Curtis distances). Samples were coloured by donor identity and include all preservation conditions and dilutions (*n*=140, 11 faecal samples and 129 cultured fraction samples). **C** Variables contributing most to compositional variation in the cultured fractions (dbRDA, genus-level Bray-Curtis distances), either independently (univariate effect sizes in dark blue/grey) or in a multivariate model (cumulative effect sizes in light blue/grey), bars in blue are significant, in grey not significant *p*<0.001

A first comparison across preservation conditions was based on the enumeration of colony-forming units (CFU) recovered from dilution plating of post-preservation samples on mGAM. Between conditions, recovery rates ranged from 1.1 × 10^7^ CFU/g to 1.9 × 10^10^ CFU/g across all dilutions (countable dilutions ranged from 10^−4^ to 10^−7^) and from 2.8 × 10^8^ CFU/g to 5.2 × 10^9^ CFU/g across the most common dilution 10^−6^ (Supplementary Table S2, Fig. 3A). For dilution 10^−6^, quantitative recovery from post-preservation samples preserved in the presence of CB medium supplemented with 5% (v/v) DMSO (condition P4) was significantly higher compared to dry preservation (P1) and preservation in CB only (P2), while not significant compared to CB supplemented with 20% (v/v) glycerol (P3) (Kruskal–Wallis with post hoc Dunn test, *p <* 0.001; *p* < 0.01, Fig. 3A).

Next, we explored to what extent preservation condition or any of the other experimental variables affected the overall community composition of the cultured fractions (i.e the harvest of the entire agar plates as illustrated in Fig. 1 after culturing post-preservation samples) harvested from the least diluted mGAM plates (*n*=129). Distance-based redundancy analysis (dbRDA) revealed that sample donor identity, the four physicochemical and microbiological sample selection parameters as well as sample dilution rate all contributed significantly to the differences in overall community composition between the cultured fractions, while sample preservation condition did not (dbRDA, adjusted *R*^2^ range = 2.7–65.5%, FDR < 0.1; Fig. 3C, Supplementary Table S3). However, only donor identity and dilution rate had a non-redundant effect on community composition (stepwise dbRDA, total *R*^2^ = 65.8%, Supplementary Table S3), with the former driving the highest effect size (Fig. 3B, C).

Bray-Curtis distances between the original faecal sample and all cultured fractions per individual also did not reveal significant differences between the preservation conditions (Kruskal-Wallis, Dunn’s paired test p >0.05) (Supplementary Table S4, Fig. 3B). Community composition analysis of original samples and their corresponding cultured fractions identified a total of 174 genera at a relative abundance threshold >0.01% of which the 20 most abundant genera together representing 75.9% of the total abundance (Supplementary Table S5). Out of these 20 genera, only 6 were detected in the cultured fractions including the genera *Bifidobacterium*, *Bacteroides* and *Phocaeicola.* They accounted for 81.4% of the total abundance, compared to 20.7% of the total average abundance in the original faecal samples. For instance, *Bifidobacterium* had an average relative abundance of only 2.9% in the uncultured faecal samples, but co-dominated cultured fractions with an average relative abundance of 39.9%. Likely, the high dominance of a few genera is driving the overall community composition in all cultured fractions and explains the lack of significance between the preservation conditions overall.

Next, we sought to identify if preservation conditions would influence total microbial richness of the recovered cultured fractions. Fitting generalized linear models (GLMs) showed that both sample dilution and richness of the uncultured source samples had a significant effect on the cultured fraction richness at the genus level (GLM, *n* = 129, *p*<0.005, Supplementary Table S6) but not at the species level. Preservation conditions did not have a significant effect on richness at any taxonomic level, independently of the fraction or only the lowest most common dilution 10^−4^ being compared (GLM, *n* = 43, *p*>0.1, Supplementary Table S6).

### Preservation conditions differentially affect recovery of specific genera

**Fig. 4:**
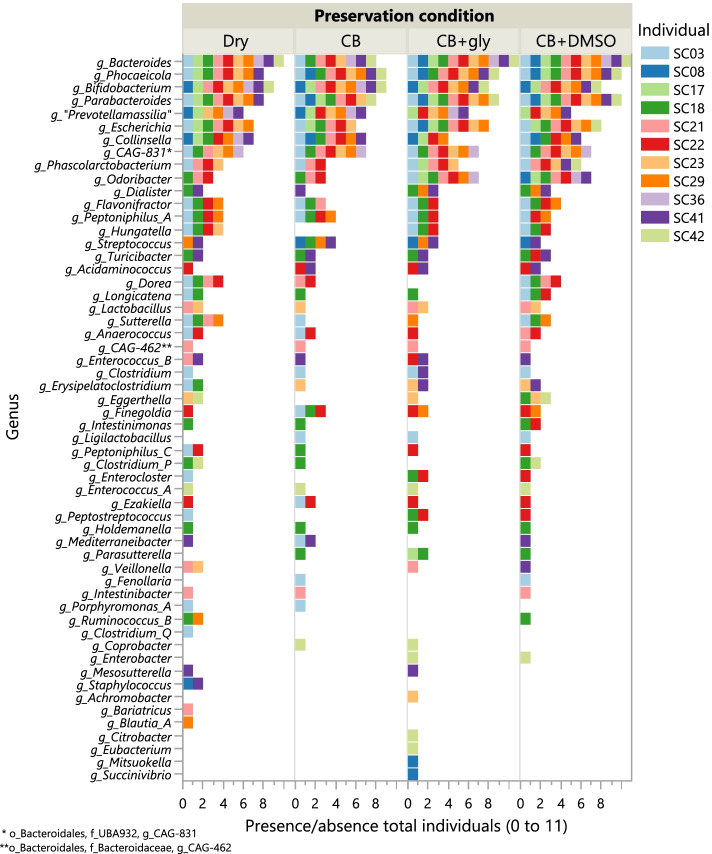
Presence-absence distribution of bacterial genera in cultured fractions (dilution 10^−4^) of each preservation condition at >0.1% abundance. The coloured bars indicate the number of individuals in which a genus was detected and each colour represents one individual

Next, in addition to comparison of overall richness, we also pursued a more narrow approach to explore whether sample preservation conditions affected anaerobic recovery of specific bacterial genera. For this purpose, we compared cultured fractions from the highest most common dilution 10^-4^, as we previously reported an effect of dilutions. In total, 45 genera were detected at >0.1% abundance in the cultured fractions of sample dilution 10^−4^ in all individuals (Fig. 4). Presence-absence analysis indicated that twenty-two of the 45 most abundant common genera were recovered in fractions from all preservation conditions, seven from three of the four preservation conditions and seven from two preservation conditions. However, the remaining nine genera were only detected in fractions from one preservation condition: five from dry (P1), two from CB + 20% (v/v) glycerol (P3) and CB + 5% (v/v) DMSO (P4) (Fig. 4). Overall, the highest number of common genera (i.e. 35/45) were recovered from sample aliquots preserved as CB + 5% (v/v) DMSO (P4) and dry (P1), followed by preservation conditions CB + 20% (v/v) glycerol (P3) (32/45), and CB only (P2) (30/45) (Fig. 4). When also taking into account relative abundances, weak significance was only found for the genus *Collinsella* which was recovered more in fractions obtained from condition CB only (P2) compared to CB + 20% (v/v) glycerol (P3), but not compared to other preservation conditions (Dunn’s test post hoc, *p* = 0.047). None of the other genera was significantly associated with a specific condition. Possible explanations for these results could be that most genera were only recovered in one or two preservation conditions from a single individual and in low abundance. Nonetheless, the observation that over half of the most common genera were not recovered in all preservation conditions indicates that different preservation methods are needed to recover specific sets of taxa. Thus, while overall richness was not significantly different between preservation conditions, differences in the recovery of individual taxa were frequently observed.

### Parallel use of different preservation conditions can increase taxonomic richness of derived culture collections

Finally, we assessed the effect of preservation conditions on the species richness of bacterial culture collections isolated from cultured fractions. Manual colony picking from plates inoculated with the highest sample dilutions resulted in a collection of 4088 isolates that were subjected to 16S rRNA gene sequencing. Sequences were obtained for 3395 isolates, resulting in a median of 82% of isolates sequenced per preservation condition per individual (range between 41 and 100%, Supplementary Table S7). Using a read length >500 bp and a sequence similarity >98% as minimal quality requirements, a total of 3008 sequenced isolates were retained for further analysis.

The 3008 isolates represented eight different phyla distributed over 54 genera and 104 species, of which the majority belonged to the phyla Actinobacteriota (38.0%) and Bacteroidota (57.1%), and at the family level, to the *Bifidobacteriaceae* (27.6%) and *Bacteroidaceae* (41.6%), respectively. Overall, culture collections with the highest richness were isolated from samples preserved as dry (P1) (36 genera and 72 species) and CB + 5% (v/v) DMSO (P4) (33 genera and 66 species). Proportional to the number sequenced isolates, the P1 strain set harboured 1.2 to 1.5 times more genera and species than other preservation conditions (Supplementary Figure S3 and Supplementary Table S8). Twenty-three genera and 43 species were exclusively found in one preservation condition: P1 (*n*=8; *n*=16), P2 (*n*=6; *n*=12), P3 (*n*=2; *n*=3) and P4 (*n*=7; *n*=12). Within the limitations of the colony selection process, these results suggest that parallel use of different sample preservation conditions can increase species and genus richness of subsequently generated culture collections.

From the 45 genera collectively recovered in cultured fractions, 23 (51.1%) were not effectively isolated in the colony picking process (Supplementary Table S9). For instance, members of the genus *Dialister* were detected in cultured fractions obtained from the lowest dilutions of sample aliquots preserved at any of the four preservation conditions but were never retrieved during the isolation process. Conversely, 30 out of the 55 genera (45.5%) represented in the entire culture collection were not detected in any of the cultured fractions (Supplementary Table S9). For example, *Akkermansia* was not detected in any cultured fraction dilution whereas 11 isolates were recovered from 4 individuals in all the tested preservation conditions (P1, *n*=8; P2–P4, *n*=1 each). Compared to the 70 genera detected at >0.01% abundance by community profiling of the uncultured faecal samples, 24 genera (32.3%) were recovered from cultured fractions and a different set of 24 genera (32.3%) were effectively isolated. Conversely, 31 genera represented in the entire culture collection were not detected by metagenomic profiling of the uncultured faecal samples. Possibly, these are low-abundant taxa that were enriched during the cultivation process (Supplementary Table 9). Comparison of taxonomic richness obtained from faecal samples, cultured fractions and culture collections indicates that sample preservation conditions, sample dilution and the semi-random colony selection process may contribute to the observation of missing taxa.

## Discussion

Preservation conditions of faecal samples aiming at subsequent isolation and cultivation of gut anaerobes have been poorly studied so far. Here, we investigated the impact of dry cryopreservation versus three cryopreservation medium formulations with or without cryoprotectant on the recovery and isolation of gut microbiota members from 11 human faecal samples. We find that the method of preservation has an important effect on the recovery and isolation of high and low abundant bacterial taxa from faecal samples. Below, we discuss several factors that might have contributed to these differences.

In any comparative study investigating the influence of sample process parameters on the preservation of microbial diversity contained in those samples, careful study design and sample selection is crucial. In this study, selection of faecal samples based on physicochemical and microbiological gradients resulted in a compositionally diverse set of samples that enabled the recovery of many different taxa. The most common preservation method of faecal samples is freezing in dry condition without addition of any medium. In our study, this preservation condition (i.e. P1) as well as the addition of CB medium supplemented with DMSO (i.e. P4) yielded the highest number of recovered common genera in cultured fractions. The addition of CB only (i.e. P2) did not seem to contribute to this effect in cultured fractions nor in isolated subcollections. While CB is a commonly used sample transport medium for maintaining the viability of enteropathogens and anaerobic bacteria, it shows a lower recovery of enteropathogens when used for cryopreservation storage [30] and is therefore better suited to preserve bacterial viability during short periods at 4°C [31]. Long-time storage at sub-zero temperatures requires addition of cryoprotectants to minimize cell damage during freezing and thawing. In our study, supplementation of CB with glycerol or DMSO was associated with the exclusive recovery of several genera from cultured fractions (Fig. 4), while no specific taxa were recovered on CB only.

A number of genera (i.e. *Blautia_A*, *Clostridium_Q*, *Enterocloster*, *Ruminococcus_B* and *Peptoniphilus_B*) were recovered only under dry preservation conditions, suggesting that CB medium and/or certain cryoprotectants might exert taxon-specific growth inhibition or toxic effects. Compared to glycerol, which has been shown to form ice crystals inside cells potentially leading to cell lysis during thawing, DMSO generally allows better recovery of a wide range of microorganisms [12, 32, 33]. This was also corroborated in the present study where CFU count-based quantitative recovery from faecal aliquots preserved in the presence of 5% (v/v) DMSO (i.e. P4) was significantly higher compared to P1 and P2 aliquots, and to a lesser extent, P3. Furthermore, two genera (i.e. *Parasutterella* and *Clostridium_P*) were exclusively recovered in cultured fractions from P4 aliquots. However, it cannot be ruled out that a concentration of 5% DMSO might inhibit recovery of certain other genera, potentially explaining why some taxa recovered exclusively under conditions P1-P3 were not detected in P4 aliquots. If logistically feasible, parallel cryopreservation of sample aliquots under multiple cryopreservation conditions such as dry and CB+DMSO storage, and/or the addition of other cryoprotectants such as trehalose [32, 34] might be recommended in order to increase cultured bacterial richness from cryopreserved faecal samples. Moreover, the addition of some compound used in preservation (e.g DMSO) may be detrimental not only to cells but also by altering gene expression and therefore transcriptomics or other omics data as already described in human cells [35] or fungi [36]. For this reason, more research may be needed to study the impact of such compound for multi-omics studies.

Also when assessing the taxonomic range among bacterial isolates semi-randomly picked from mGAM plates, the combined culture collections derived from dry cryopreservation and aliquots stored in the presence of CB+DMSO yielded the highest number of cultured species, with more taxa specifically isolated from dry cryopreservation. Evidently, the choice of culture medium to compare bacterial recovery across preservation conditions is of great importance. To keep the study logistically manageable, we restricted comparisons to only the widely used anaerobic mGAM medium previously shown to enable the growth of many gut anaerobes [3, 17, 18]. Still, 67.9% of the isolates recovered from cultured fractions in our study belonged to just three genera, i.e. *Bifidobacterium* (31.3%)*, Bacteroides* (27.0%) and *Phocaeicola* (9.6%), while in uncultured samples these taxa had a collated relative abundance of only 20.7% (2.9%, 7.0% and 10.8% respectively). Apart from the possibility that part of the cells detected in community sequencing might be dead, damaged or metabolically inactive, it is likely that the nutritional conditions of the mGAM medium favour recovery of a few specific taxa but inhibit or lack an essential growth factor of other highly abundant gut members that remained undetected in cultured fractions. Future study designs including nutritional supplementation of mGAM and/or the use of additional media can be useful to check to what extent the recovery of major missing taxa such as *Faecalibacterium* would have been affected differently by the tested preservation conditions. Next to preservation condition and medium, however, our study shows that also the sample dilution rate co-determines the species richness recovered during isolation. This might be especially relevant for low-abundant species that can be washed out during the dilution process and/or of which the few remaining cells are overgrown by fast-growing species. In addition, it should be kept in mind that other limitations of agar-based isolation approaches such as the overgrowth of slow-growing species by faster-growing ones and the inability to pick microscopically small colonies might further distort the investigator’s view on the culturable diversity of a sample. Single-cell based strategies, ranging from dilution-to-extinction to microfluidic single-cell droplet technologies, suffer much less from these shortcomings and are emerging at a rapid pace [37].

## Conclusion

In this study, community profiling of faecal samples and cultured fractions was combined with 16S rRNA gene sequence identification of isolated colonies. Not only did this confirm the complementarity of culturing and metagenomics approaches to cover the largest taxonomic spectrum possible [38], but it also enabled us to investigate the potential impact of sample cryopreservation conditions at multiple levels. We demonstrated that parallel use of multiple faecal cryopreservation conditions can clearly benefit the taxonomic depth of gut culturomics efforts. Therefore, the current practice of cryopreserving stool samples in dry conditions may need to be complemented with other preservation medium conditions such as CB + DMSO to increase the species diversity of gut-associated culture collections. Moreover, we show that a sample selection strategy based on physicochemical and microbiological gradients is another key contributor in maximizing the recovery of a phylogenetically diverse set of bacterial taxa from human stool samples.

## Supplementary Information

**Additional file 1: Figure S1**: Distribution of gradients among the 51 samples, selected samples are coloured.

**Additional file 2: Figure S2**: Spearman correlation heatmap of the faecal variables, no significance was observed.

**Additional file 3: Figure S3**: Proportion of taxa per total sequence per preservation conditions at species and genus level.

**Additional file 4: Supplementary Table S1**: Gradients in physicochemical and biological parameters of the 11 donor samples.

**Additional file 5: Supplementary Table S2**: Cell counts of the cultured fractions for each individual and each preservation condition.

**Additional file 6: Supplementary Table S3**: Cumulative and independent contribution of metadata variables to community variation of the cultured fractions (dbRDA and stepwise dbRDA; FDR by Benjamini-Hochberg) in the cohort (*n*=129). Cumulative explanatory power and significance level of the included variables are reported.

**Additional file 7: Supplementary Table S4**: Bray-curtis dissimilarity distances for each sample (*n*=129) to its respective feacal sample.

**Additional file 8: Supplementary Table S5**: Relative abundances of genera detected in feacal and cultured fractions samples in culture-independent (faecal) and cultured fractions per preservation conditions (P1 to P4).

**Additional file 9: Supplementary Table S6**: Associations between richness in the cultured fractions and the original richness, the preservation conditions and dilutions (*n*=129).

**Additional file 10: Supplementary Table S7**: Summary of the number of isolates and the number of recovered sequences from 16S rDNA sequencing of the isolates per individual and preservation condition.

**Additional file 11: Supplementary Table S8**: Proportion of taxa per total sequences for each preservation condition at genus and species level.

**Additional file 12: Supplementary Table S9**: Presence/absence of genera between culture-independent (feacal samples), cultured fractions and isolates, detection threshold >0.01%.

## Data Availability

The data for this study have been deposited in the European Nucleotide Archive (ENA) at EMBL-EBI under accession number PRJEB49125 (https://www.ebi.ac.uk/ena/browser/view/PRJEB49125).
