# Supplementary figures and images for: Effect of cryopreservation medium conditions on growth and isolation of gut anaerobes from human faecal samples
Source: Microbiome. 2022 May 30;10:80. doi: 10.1186/s40168-022-01267-2 (PMC9150342; doi:10.1186/s40168-022-01267-2)

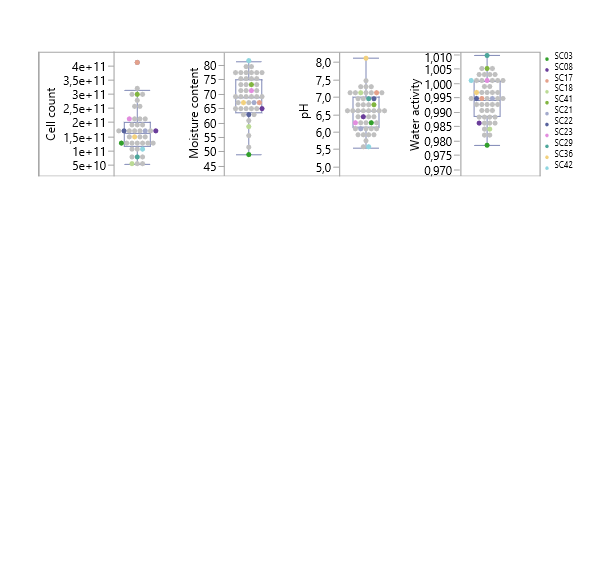

Supplement: Supplementary file 2 — Additional file 1: Figure S1: Distribution of gradients among the 51 samples, selected samples are coloured. [file 40168_2022_1267_MOESM2_ESM.docx]

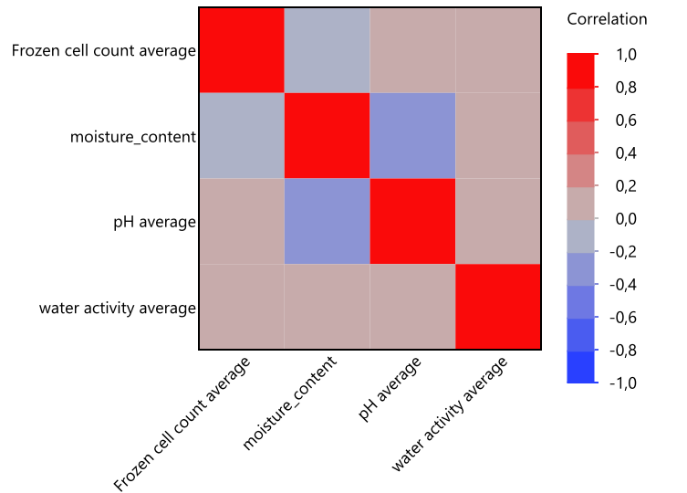

Supplement: Supplementary file 3 — Additional file 2: Figure S2: Spearman correlation heatmap of the faecal variables, no significance was observed. [file 40168_2022_1267_MOESM3_ESM.docx]

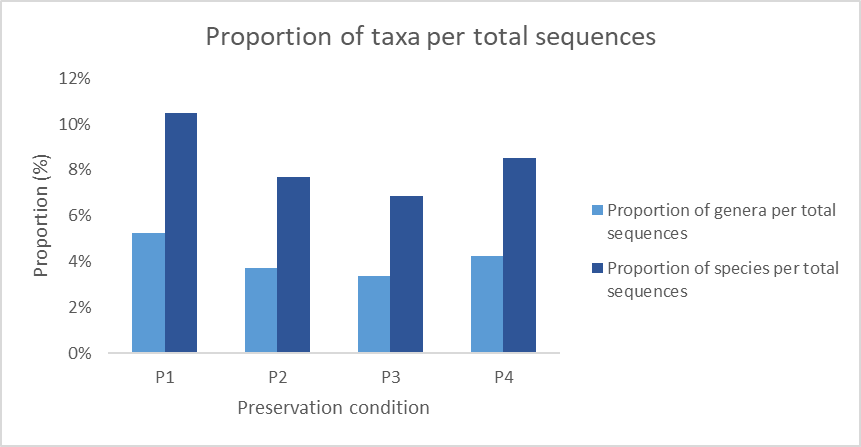

Supplement: Supplementary file 4 — Additional file 3: Figure S3: Proportion of taxa per total sequence per preservation conditions at species and genus level. [file 40168_2022_1267_MOESM4_ESM.docx]
