# Supplementary material for: Effect of cryopreservation medium conditions on growth and isolation of gut anaerobes from human faecal samples
Source: Microbiome. 2022 May 30;10:80. doi: 10.1186/s40168-022-01267-2 (PMC9150342; doi:10.1186/s40168-022-01267-2)
Supplement: Supplementary file 5 — Additional file 4: Supplementary Table S1: Gradients in physicochemical and biological parameters of the 11 donor samples. [file 40168_2022_1267_MOESM5_ESM.docx]

| **Supplementary Table S1: Gradients in physicochemical and biological parameters of the 11 donor samples.** | |
| --- | --- |
| **Parameter** | **Range** |
| **pH** | 5.58 - 8.15 |
| **Water activity** | 0.973 - 1.000 |
| **Frozen cell count** | 4.21x10^10^ - 4.04x10^11^ cells/g |
| **Moisture content** | 49.8 - 81.9 % |
