# Supplementary material for: Effect of cryopreservation medium conditions on growth and isolation of gut anaerobes from human faecal samples
Source: Microbiome. 2022 May 30;10:80. doi: 10.1186/s40168-022-01267-2 (PMC9150342; doi:10.1186/s40168-022-01267-2)
Supplement: Supplementary file 6 — Additional file 5: Supplementary Table S2: Cell counts of the cultured fractions for each individual and each preservation condition. [file 40168_2022_1267_MOESM6_ESM.docx]

| **Supplementary Table S2: Cell counts of the cultured fractions for each individual and each preservation condition.** | | | | |
| --- | --- | --- | --- | --- |
| **Sample_ID** | **Preservation** | **Dilution** | **Replicate** | **Cultured_fraction_cell_count** |
| SC03 | P1 | D-6 | 1 | 5.36E+09 |
| SC03 | P1 | D-6 | 2 | 5.42E+09 |
| SC03 | P1 | D-6 | 3 | 5.62E+09 |
| SC03 | P1 | D-7 | 1 | 7.40E+08 |
| SC03 | P1 | D-7 | 2 | 6.00E+08 |
| SC03 | P1 | D-7 | 3 | 8.20E+08 |
| SC03 | P2 | D-5 | 1 | 5.28E+09 |
| SC03 | P2 | D-5 | 2 | 4.88E+09 |
| SC03 | P2 | D-5 | 3 | 5.06E+09 |
| SC03 | P2 | D-6 | 1 | 1.34E+09 |
| SC03 | P2 | D-6 | 2 | 1.20E+09 |
| SC03 | P2 | D-6 | 3 | 1.04E+09 |
| SC03 | P3 | D-6 | 1 | 1.72E+09 |
| SC03 | P3 | D-6 | 2 | 1.76E+09 |
| SC03 | P3 | D-6 | 3 | 1.62E+09 |
| SC03 | P4 | D-6 | 1 | 3.80E+09 |
| SC03 | P4 | D-6 | 2 | 3.20E+09 |
| SC03 | P4 | D-6 | 3 | 2.92E+09 |
| SC03 | P4 | D-7 | 1 | 4.80E+09 |
| SC03 | P4 | D-7 | 2 | 4.60E+09 |
| SC03 | P4 | D-7 | 3 | 6.00E+09 |
| SC18 | P1 | D-6 | 1 | 9.20E+08 |
| SC18 | P1 | D-6 | 2 | 9.80E+08 |
| SC18 | P1 | D-6 | 3 | 9.20E+08 |
| SC18 | P2 | D-5 | 1 | 4.02E+09 |
| SC18 | P2 | D-5 | 2 | 3.40E+09 |
| SC18 | P2 | D-5 | 3 | 3.54E+09 |
| SC18 | P2 | D-6 | 1 | 8.60E+08 |
| SC18 | P2 | D-6 | 2 | 7.20E+08 |
| SC18 | P2 | D-6 | 3 | 7.80E+08 |
| SC18 | P3 | D-6 | 1 | 1.90E+09 |
| SC18 | P3 | D-6 | 2 | 1.80E+09 |
| SC18 | P3 | D-6 | 3 | 1.88E+09 |
| SC18 | P3 | D-7 | 1 | 3.20E+09 |
| SC18 | P3 | D-7 | 2 | 2.00E+09 |
| SC18 | P3 | D-7 | 3 | 3.20E+09 |
| SC18 | P4 | D-6 | 1 | 2.46E+09 |
| SC18 | P4 | D-6 | 2 | 2.02E+09 |
| SC18 | P4 | D-6 | 3 | 3.08E+09 |
| SC22 | P1 | D-6 | 1 | 1.38E+09 |
| SC22 | P1 | D-6 | 2 | 1.54E+09 |
| SC22 | P1 | D-6 | 3 | 1.26E+09 |
| SC22 | P2 | D-5 | 1 | 2.66E+09 |
| SC22 | P2 | D-5 | 2 | 2.38E+09 |
| SC22 | P2 | D-5 | 3 | 2.28E+09 |
| SC22 | P2 | D-6 | 1 | 4.40E+08 |
| SC22 | P2 | D-6 | 2 | 2.80E+08 |
| SC22 | P2 | D-6 | 3 | 5.20E+08 |
| SC22 | P3 | D-6 | 1 | 7.60E+08 |
| SC22 | P3 | D-6 | 2 | 8.60E+08 |
| SC22 | P3 | D-6 | 3 | 8.80E+08 |
| SC22 | P4 | D-6 | 1 | 8.00E+08 |
| SC22 | P4 | D-6 | 2 | 1.04E+09 |
| SC22 | P4 | D-6 | 3 | 9.00E+08 |
| SC08 | P1 | D-6 | 1 | 1.40E+09 |
| SC08 | P1 | D-6 | 2 | 2.00E+09 |
| SC08 | P1 | D-6 | 3 | 1.42E+09 |
| SC08 | P2 | D-6 | 1 | 3.24E+09 |
| SC08 | P2 | D-6 | 2 | 4.02E+09 |
| SC08 | P2 | D-6 | 3 | 3.72E+09 |
| SC08 | P2 | D-7 | 1 | 5.40E+09 |
| SC08 | P2 | D-7 | 2 | 2.80E+09 |
| SC08 | P2 | D-7 | 3 | 6.40E+09 |
| SC08 | P3 | D-6 | 1 | 3.64E+09 |
| SC08 | P3 | D-6 | 2 | 3.62E+09 |
| SC08 | P3 | D-6 | 3 | 3.06E+09 |
| SC08 | P3 | D-7 | 1 | 5.60E+09 |
| SC08 | P3 | D-7 | 2 | 2.40E+09 |
| SC08 | P3 | D-7 | 3 | 4.00E+09 |
| SC08 | P4 | D-6 | 1 | 3.88E+09 |
| SC08 | P4 | D-6 | 2 | 4.24E+09 |
| SC08 | P4 | D-6 | 3 | 3.98E+09 |
| SC08 | P4 | D-7 | 1 | 3.80E+09 |
| SC08 | P4 | D-7 | 2 | 3.40E+09 |
| SC08 | P4 | D-7 | 3 | 3.80E+09 |
| SC36 | P1 | D-6 | 1 | 1.46E+09 |
| SC36 | P1 | D-6 | 2 | 1.80E+09 |
| SC36 | P1 | D-6 | 3 | 1.64E+09 |
| SC36 | P1 | D-7 | 1 | 2.40E+08 |
| SC36 | P1 | D-7 | 2 | 1.80E+08 |
| SC36 | P1 | D-7 | 3 | 1.40E+08 |
| SC36 | P2 | D-5 | 1 | 4.98E+09 |
| SC36 | P2 | D-5 | 2 | 5.72E+09 |
| SC36 | P2 | D-5 | 3 | 4.64E+09 |
| SC36 | P2 | D-6 | 1 | 9.60E+08 |
| SC36 | P2 | D-6 | 2 | 9.20E+08 |
| SC36 | P2 | D-6 | 3 | 8.60E+08 |
| SC36 | P3 | D-5 | 1 | 2.94E+07 |
| SC36 | P3 | D-5 | 2 | 3.18E+07 |
| SC36 | P3 | D-5 | 3 | 2.78E+07 |
| SC36 | P3 | D-6 | 1 | 1.00E+09 |
| SC36 | P3 | D-6 | 2 | 1.16E+09 |
| SC36 | P3 | D-6 | 3 | 1.20E+09 |
| SC36 | P4 | D-5 | 1 | 3.26E+07 |
| SC36 | P4 | D-5 | 2 | 2.90E+07 |
| SC36 | P4 | D-5 | 3 | 3.20E+07 |
| SC36 | P4 | D-6 | 1 | 1.36E+09 |
| SC36 | P4 | D-6 | 2 | 1.34E+09 |
| SC36 | P4 | D-6 | 3 | 1.40E+09 |
| SC21 | P1 | D-5 | 1 | 3.78E+08 |
| SC21 | P1 | D-5 | 2 | 3.30E+08 |
| SC21 | P1 | D-5 | 3 | 2.80E+08 |
| SC21 | P1 | D-6 | 1 | 5.60E+08 |
| SC21 | P1 | D-6 | 2 | 8.00E+08 |
| SC21 | P1 | D-6 | 3 | 1.28E+09 |
| SC21 | P2 | D-6 | 1 | 2.82E+09 |
| SC21 | P2 | D-6 | 2 | 3.26E+09 |
| SC21 | P2 | D-6 | 3 | 3.30E+09 |
| SC21 | P2 | D-7 | 1 | 6.00E+09 |
| SC21 | P2 | D-7 | 2 | 7.40E+09 |
| SC21 | P2 | D-7 | 3 | 4.20E+09 |
| SC21 | P3 | D-6 | 1 | 4.86E+09 |
| SC21 | P3 | D-6 | 2 | 4.88E+09 |
| SC21 | P3 | D-6 | 3 | 4.22E+09 |
| SC21 | P3 | D-7 | 1 | 4.00E+09 |
| SC21 | P3 | D-7 | 2 | 2.00E+09 |
| SC21 | P3 | D-7 | 3 | 3.40E+09 |
| SC21 | P4 | D-6 | 1 | 3.40E+09 |
| SC21 | P4 | D-6 | 2 | 3.58E+09 |
| SC21 | P4 | D-6 | 3 | 3.80E+09 |
| SC21 | P4 | D-7 | 1 | 7.00E+09 |
| SC21 | P4 | D-7 | 2 | 5.60E+09 |
| SC21 | P4 | D-7 | 3 | 4.00E+09 |
| SC17 | P1 | D-6 | 1 | 1.34E+09 |
| SC17 | P1 | D-6 | 2 | 1.52E+09 |
| SC17 | P1 | D-6 | 3 | 1.24E+09 |
| SC17 | P2 | D-6 | 1 | 2.38E+09 |
| SC17 | P2 | D-6 | 2 | 1.96E+09 |
| SC17 | P2 | D-6 | 3 | 2.28E+09 |
| SC17 | P2 | D-7 | 1 | 5.20E+09 |
| SC17 | P2 | D-7 | 2 | 3.60E+09 |
| SC17 | P2 | D-7 | 3 | 3.20E+09 |
| SC17 | P3 | D-6 | 1 | 5.60E+08 |
| SC17 | P3 | D-6 | 2 | 6.80E+08 |
| SC17 | P3 | D-6 | 3 | 6.60E+08 |
| SC17 | P4 | D-6 | 1 | 1.34E+09 |
| SC17 | P4 | D-6 | 2 | 1.82E+09 |
| SC17 | P4 | D-6 | 3 | 1.68E+09 |
| SC17 | P4 | D-7 | 1 | 4.00E+09 |
| SC17 | P4 | D-7 | 2 | 4.20E+09 |
| SC17 | P4 | D-7 | 3 | 2.00E+09 |
| SC23 | P1 | D-6 | 1 | 1.60E+09 |
| SC23 | P1 | D-6 | 2 | 2.16E+09 |
| SC23 | P1 | D-6 | 3 | 1.70E+09 |
| SC23 | P2 | D-6 | 1 | 3.68E+09 |
| SC23 | P2 | D-6 | 2 | 4.44E+09 |
| SC23 | P2 | D-6 | 3 | 3.94E+09 |
| SC23 | P2 | D-7 | 1 | 4.80E+09 |
| SC23 | P2 | D-7 | 2 | 4.20E+09 |
| SC23 | P2 | D-7 | 3 | 4.60E+09 |
| SC23 | P3 | D-7 | 1 | 1.22E+10 |
| SC23 | P3 | D-7 | 2 | 1.04E+10 |
| SC23 | P3 | D-7 | 3 | 1.08E+10 |
| SC23 | P4 | D-7 | 1 | 8.60E+09 |
| SC23 | P4 | D-7 | 2 | 1.18E+10 |
| SC41 | P1 | D-7 | 1 | 7.80E+08 |
| SC41 | P1 | D-7 | 2 | 7.20E+08 |
| SC41 | P1 | D-7 | 3 | 7.60E+08 |
| SC41 | P2 | D-7 | 1 | 7.40E+09 |
| SC41 | P2 | D-7 | 2 | 9.20E+09 |
| SC41 | P2 | D-7 | 3 | 9.00E+09 |
| SC41 | P3 | D-7 | 1 | 1.06E+10 |
| SC41 | P3 | D-7 | 2 | 9.00E+09 |
| SC41 | P3 | D-7 | 3 | 1.00E+10 |
| SC41 | P4 | D-7 | 1 | 1.90E+10 |
| SC41 | P4 | D-7 | 2 | 1.66E+10 |
| SC41 | P4 | D-7 | 3 | 1.40E+10 |
| SC42 | P1 | D-5 | 1 | 3.80E+07 |
| SC42 | P1 | D-5 | 2 | 2.60E+07 |
| SC42 | P1 | D-5 | 3 | 3.40E+07 |
| SC42 | P2 | D-4 | 1 | 1.78E+07 |
| SC42 | P2 | D-4 | 2 | 3.52E+07 |
| SC42 | P2 | D-4 | 3 | 1.44E+07 |
| SC42 | P3 | D-4 | 1 | 1.14E+07 |
| SC42 | P3 | D-4 | 2 | 1.12E+07 |
| SC42 | P3 | D-4 | 3 | 1.36E+07 |
| SC42 | P4 | D-4 | 1 | 1.28E+07 |
| SC42 | P4 | D-4 | 2 | 1.44E+07 |
| SC42 | P4 | D-4 | 3 | 1.28E+07 |
| SC29 | P1 | D-5 | 1 | 3.98E+08 |
| SC29 | P1 | D-5 | 2 | 3.12E+08 |
| SC29 | P1 | D-5 | 3 | 4.16E+08 |
| SC29 | P1 | D-6 | 1 | 4.60E+08 |
| SC29 | P1 | D-6 | 2 | 5.00E+08 |
| SC29 | P1 | D-6 | 3 | 3.60E+08 |
| SC29 | P2 | D-5 | 1 | 4.78E+09 |
| SC29 | P2 | D-5 | 2 | 5.96E+09 |
| SC29 | P2 | D-5 | 3 | 6.46E+09 |
| SC29 | P2 | D-6 | 1 | 1.58E+09 |
| SC29 | P2 | D-6 | 2 | 5.00E+08 |
| SC29 | P2 | D-6 | 3 | 8.80E+08 |
| SC29 | P3 | D-6 | 1 | 3.28E+09 |
| SC29 | P3 | D-6 | 2 | 3.00E+09 |
| SC29 | P3 | D-6 | 3 | 3.38E+09 |
| SC29 | P3 | D-7 | 1 | 3.40E+09 |
| SC29 | P3 | D-7 | 2 | 3.20E+09 |
| SC29 | P3 | D-7 | 3 | 2.80E+09 |
| SC29 | P4 | D-6 | 1 | 4.10E+09 |
| SC29 | P4 | D-6 | 2 | 3.82E+09 |
| SC29 | P4 | D-6 | 3 | 3.86E+09 |
| SC29 | P4 | D-7 | 1 | 9.80E+09 |
| SC29 | P4 | D-7 | 2 | 8.60E+09 |
| SC29 | P4 | D-7 | 3 | 8.80E+09 |
