# Supplementary material for: Effect of cryopreservation medium conditions on growth and isolation of gut anaerobes from human faecal samples
Source: Microbiome. 2022 May 30;10:80. doi: 10.1186/s40168-022-01267-2 (PMC9150342; doi:10.1186/s40168-022-01267-2)
Supplement: Supplementary file 7 — Additional file 6: Supplementary Table S3: Cumulative and independent contribution of metadata variables to community variation of the cultured fractions (dbRDA and stepwise dbRDA; FDR by Benjamini-Hochberg) in the cohort (n=129). Cumulative explanatory power and significance level of the included variables are reported. [file 40168_2022_1267_MOESM7_ESM.docx]

| **Supplementary Table S3: Cumulative and independent contribution of metadata variables to community variation of the cultured fractions (dbRDA and stepwise dbRDA; FDR by Benjamini-Hochberg) in the cohort (n=129). Cumulative explanatory power and significance level of the included variables are reported.** | | | | | | |
| --- | --- | --- | --- | --- | --- | --- |
|  |  |  |  |  |  |  |
| dbRDA assuming variable independence | | | | |  |  |
| **Metadata** | **Fmodel** | **R2** | **p-value** | **BH adj p-value** | |  |
| Preservation | 1.03953 | 0.027328 | 0.407592 | 0.407592 |  |  |
| Dilution | 1.889848 | 0.048595 | 0.015984 | 0.018648 |  |  |
| Frozen.cell.count.average | 18.44472 | 0.581949 | 0.000999 | 0.001399 |  |  |
| moisture_content | 18.33503 | 0.139605 | 0.000999 | 0.001399 |  |  |
| pH.average | 7.676318 | 0.063611 | 0.000999 | 0.001399 |  |  |
| water.activity.average | 14.57956 | 0.114278 | 0.000999 | 0.001399 |  |  |
| Individual | 22.20622 | 0.655575 | 0.000999 | 0.001399 |  |  |
|  |  |  |  |  |  |  |
| stepwise dbRDA | | | | |  |  |
| **Metadata** | **R2.adj** | **Df** | **AIC** | **F** | **Pr..F.** |  |
| Individual | 0.63291 | 9 | 339.5874 | 1.78E+01 | 1.00E-04 |  |
| Dilution | 0.6579 | 3 | 336.7598 | 2.71E+00 | 1.00E-04 |  |
