# Supplementary material for: Effect of cryopreservation medium conditions on growth and isolation of gut anaerobes from human faecal samples
Source: Microbiome. 2022 May 30;10:80. doi: 10.1186/s40168-022-01267-2 (PMC9150342; doi:10.1186/s40168-022-01267-2)
Supplement: Supplementary file 8 — Additional file 7: Supplementary Table S4: Bray-curtis dissimilarity distances for each sample (n=129) to its respective feacal sample. [file 40168_2022_1267_MOESM8_ESM.docx]

| **Supplementary Table S4: Bray-curtis dissimilarity distances for each sample (n=129) to its respective feacal sample.** | |
| --- | --- |
| **Sample** | **Bray-Curtis distance to faecal sample** |
| SC03_P1_10.4 | 0.930327869 |
| SC03_P1_10.5 | 0.927595628 |
| SC03_P1_10.6 | 0.949453552 |
| SC03_P2_10.4 | 0.926229508 |
| SC03_P2_10.5 | 0.930327869 |
| SC03_P2_10.6 | 0.93579235 |
| SC03_P3_10.4 | 0.943989071 |
| SC03_P3_10.5 | 0.952185792 |
| SC03_P3_10.6 | 0.941256831 |
| SC03_P4_10.4 | 0.942622951 |
| SC03_P4_10.5 | 0.93715847 |
| SC03_P4_10.6 | 0.959016393 |
| SC08_P1_10.4 | 0.961748634 |
| SC08_P1_10.5 | 0.963114754 |
| SC08_P1_10.6 | 0.931693989 |
| SC08_P2_10.4 | 0.93442623 |
| SC08_P2_10.5 | 0.922131148 |
| SC08_P2_10.6 | 0.93852459 |
| SC08_P3_10.4 | 0.908469945 |
| SC08_P3_10.5 | 0.909836066 |
| SC08_P3_10.6 | 0.908469945 |
| SC08_P4_10.4 | 0.908469945 |
| SC08_P4_10.5 | 0.908469945 |
| SC08_P4_10.6 | 0.908469945 |
| SC17_P1_10.4 | 0.983606557 |
| SC17_P1_10.5 | 0.956284153 |
| SC17_P1_10.6 | 0.855191257 |
| SC17_P2_10.4 | 0.975409836 |
| SC17_P2_10.5 | 0.953551913 |
| SC17_P2_10.6 | 0.953551913 |
| SC17_P3_10.3 | 0.960382514 |
| SC17_P3_10.4 | 0.946721311 |
| SC17_P3_10.5 | 0.819672131 |
| SC17_P4_10.4 | 0.965846995 |
| SC17_P4_10.5 | 0.821038251 |
| SC17_P4_10.6 | 0.819672131 |
| SC18_P1_10.3 | 0.781420765 |
| SC18_P1_10.4 | 0.781420765 |
| SC18_P1_10.5 | 0.786885246 |
| SC18_P2_10.3 | 0.771857923 |
| SC18_P2_10.4 | 0.782786885 |
| SC18_P2_10.5 | 0.782786885 |
| SC18_P3_10.4 | 0.785519126 |
| SC18_P3_10.5 | 0.786885246 |
| SC18_P3_10.6 | 0.789617486 |
| SC18_P4_10.4 | 0.785519126 |
| SC18_P4_10.5 | 0.777322404 |
| SC18_P4_10.6 | 0.788251366 |
| SC21_P1_10.3 | 0.745901639 |
| SC21_P1_10.4 | 0.781420765 |
| SC21_P1_10.5 | 0.762295082 |
| SC21_P2_10.4 | 0.743169399 |
| SC21_P2_10.5 | 0.75136612 |
| SC21_P2_10.6 | 0.773224044 |
| SC21_P3_10.4 | 0.819672131 |
| SC21_P3_10.5 | 0.784153005 |
| SC21_P3_10.6 | 0.767759563 |
| SC21_P4_10.4 | 0.740437158 |
| SC21_P4_10.5 | 0.760928962 |
| SC21_P4_10.6 | 0.75273224 |
| SC22_P1_10.3 | 0.841530055 |
| SC22_P1_10.4 | 0.81557377 |
| SC22_P1_10.5 | 0.800546448 |
| SC22_P2_10.3 | 0.834699454 |
| SC22_P2_10.4 | 0.801912568 |
| SC22_P2_10.5 | 0.841530055 |
| SC22_P3_10.3 | 0.844262295 |
| SC22_P3_10.4 | 0.837431694 |
| SC22_P3_10.5 | 0.893442623 |
| SC22_P4_10.3 | 0.834699454 |
| SC22_P4_10.4 | 0.838797814 |
| SC22_P4_10.5 | 0.897540984 |
| SC23_P1_10.4 | 0.919398907 |
| SC23_P1_10.5 | 0.769125683 |
| SC23_P1_10.6 | 0.628415301 |
| SC23_P2_10.4 | 0.946721311 |
| SC23_P2_10.5 | 0.740437158 |
| SC23_P2_10.6 | 0.612021858 |
| SC23_P3_10.4 | 0.952185792 |
| SC23_P3_10.5 | 0.607923497 |
| SC23_P3_10.6 | 0.754098361 |
| SC23_P4_10.4 | 0.871584699 |
| SC23_P4_10.5 | 0.81147541 |
| SC23_P4_10.6 | 0.607923497 |
| SC29_P1_10.3 | 0.923497268 |
| SC29_P1_10.4 | 0.912568306 |
| SC29_P1_10.5 | 0.922131148 |
| SC29_P2_10.3 | 0.924863388 |
| SC29_P2_10.4 | 0.928961749 |
| SC29_P2_10.5 | 0.928961749 |
| SC29_P3_10.4 | 0.911202186 |
| SC29_P3_10.5 | 0.920765027 |
| SC29_P3_10.6 | 0.923497268 |
| SC29_P4_10.4 | 0.915300546 |
| SC29_P4_10.5 | 0.922131148 |
| SC29_P4_10.6 | 0.972677596 |
| SC36_P1_10.4 | 0.823770492 |
| SC36_P1_10.5 | 0.81557377 |
| SC36_P1_10.6 | 0.819672131 |
| SC36_P2_10.4 | 0.823770492 |
| SC36_P2_10.5 | 0.825136612 |
| SC36_P2_10.6 | 0.826502732 |
| SC36_P3_10.4 | 0.826502732 |
| SC36_P3_10.5 | 0.849726776 |
| SC36_P3_10.6 | 0.81420765 |
| SC36_P4_10.4 | 0.836065574 |
| SC36_P4_10.5 | 0.846994536 |
| SC36_P4_10.6 | 0.844262295 |
| SC41_P1_10.4 | 0.924863388 |
| SC41_P1_10.5 | 0.922131148 |
| SC41_P1_10.6 | 0.81147541 |
| SC41_P2_10.4 | 0.931693989 |
| SC41_P2_10.5 | 0.870218579 |
| SC41_P2_10.6 | 0.864754098 |
| SC41_P3_10.4 | 0.905737705 |
| SC41_P3_10.5 | 0.885245902 |
| SC41_P3_10.6 | 0.801912568 |
| SC41_P4_10.4 | 0.786885246 |
| SC41_P4_10.5 | 0.785519126 |
| SC41_P4_10.6 | 0.785519126 |
| SC42_P1_10.3 | 0.978142077 |
| SC42_P1_10.4 | 0.965846995 |
| SC42_P1_10.5 | 0.825136612 |
| SC42_P2_10.3 | 0.979508197 |
| SC42_P2_10.4 | 0.856557377 |
| SC42_P3_10.3 | 0.825136612 |
| SC42_P3_10.4 | 0.825136612 |
| SC42_P4_10.3 | 0.816939891 |
| SC42_P4_10.4 | 0.825136612 |
