# Supplementary material for: Effect of cryopreservation medium conditions on growth and isolation of gut anaerobes from human faecal samples
Source: Microbiome. 2022 May 30;10:80. doi: 10.1186/s40168-022-01267-2 (PMC9150342; doi:10.1186/s40168-022-01267-2)
Supplement: Supplementary file 9 — Additional file 8: Supplementary Table S5: Relative abundances of genera detected in feacal and cultured fractions samples in culture-independent (faecal) and cultured fractions per preservation conditions (P1 to P4). [file 40168_2022_1267_MOESM9_ESM.docx]

| **Supplementary Table S5: Relative abundances of genera detected in feacal and cultured fractions samples in culture-independent (faecal) and cultured fractions per preservation conditions (P1 to P4).** | | | | | | |
| --- | --- | --- | --- | --- | --- | --- |
| **Genus** | **Faecal** | **P1** | **P2** | **P3** | **P4** | **Average (P1-P4)** |
| *Faecalibacterium* | 0.187206 | 0 | 0 | 0 | 0 | 0 |
| *Phocaeicola* | 0.107986 | 0.092641 | 0.143843 | 0.16993 | 0.248975 | 0.163847366 |
| *Bacteroides* | 0.07046 | 0.203044 | 0.21519 | 0.286128 | 0.30372 | 0.252020566 |
| *Agathobacter* | 0.053805 | 0 | 0 | 0 | 0 | 0 |
| *Blautia_A* | 0.049818 | 0 | 0 | 0 | 0 | 0 |
| *Roseburia* | 0.03307 | 0 | 0 | 0 | 0 | 0 |
| *Gemmiger* | 0.031871 | 0 | 0 | 0 | 0 | 0 |
| *Bifidobacterium* | 0.028796 | 0.48798 | 0.508188 | 0.333096 | 0.264959 | 0.398555798 |
| *Alistipes* | 0.02644 | 0 | 0 | 0 | 0 | 0 |
| *Prevotella* | 0.02608 | 0 | 0 | 0 | 0 | 0 |
| *Ruminococcus_E* | 0.021203 | 0 | 0 | 0 | 0 | 0 |
| *Erysipelatoclostridium* | 0.021128 | 0 | 0 | 0.000331 | 0.00061 | 0.00023541 |
| *Dorea* | 0.015884 | 0.000152 | 0.000103 | 0 | 0.00029 | 0.00013624 |
| *Anaerostipes* | 0.013717 | 0 | 0 | 0 | 0 | 0 |
| *Fusicatenibacter* | 0.013381 | 0 | 0 | 0 | 0 | 0 |
| *Lachnospira* | 0.012774 | 0 | 0 | 0 | 0 | 0 |
| *Acetatifactor* | 0.012604 | 0 | 0 | 0 | 0 | 0 |
| *Anaerobutyricum* | 0.012115 | 0 | 0 | 0 | 0 | 0 |
| *Coprococcus* | 0.010794 | 0 | 0 | 0 | 0 | 0 |
| *CAG-831* | 0.009709 | 0.001289 | 0.002114 | 0.004004 | 0.002408 | 0.002453666 |
| *Prevotellamassilia* | 0.008309 | 0.044709 | 0.04831 | 0.035389 | 0.030811 | 0.039804844 |
| *Mediterraneibacter* | 0.007576 | 0 | 0.000106 | 0 | 0 | 2.65751E-05 |
| *uc_f_Lachnospiraceae* | 0.006905 | 0 | 0 | 0 | 0 | 0 |
| *Parabacteroides* | 0.00677 | 0.016393 | 0.016792 | 0.031922 | 0.033566 | 0.024668402 |
| *Dialister* | 0.006448 | 0.000318 | 0.000325 | 0.001047 | 0.002648 | 0.001084682 |
| *UBA11471* | 0.006308 | 0 | 0 | 0 | 0 | 0 |
| *ER4* | 0.006055 | 0 | 0 | 0 | 0 | 0 |
| *UMGS1994* | 0.006032 | 0 | 0 | 0 | 0 | 0 |
| *Bariatricus* | 0.00586 | 0 | 0 | 0 | 0 | 0 |
| *Enterococcus_B* | 0 | 0.003734 | 0.003711 | 0.004111 | 0.002098 |  |
| *Methanobrevibacter_A* | 0.005791 | 0 | 0 | 0 | 0 | 0 |
| *Ruminococcus_D* | 0.005269 | 0 | 0 | 0 | 0 | 0 |
| *Collinsella* | 0.005177 | 0.023754 | 0.007363 | 0.00155 | 0.003318 | 0.008996412 |
| *CAG-83* | 0.004794 | 0 | 0 | 0 | 0 | 0 |
| *UBA7173* | 0.004688 | 0 | 0 | 0 | 0 | 0 |
| *Agathobaculum* | 0.004545 | 0 | 0 | 0 | 0 | 0 |
| *Akkermansia* | 0.004391 | 0 | 0 | 0 | 0 | 0 |
| *Streptococcus* | 0.004371 | 0.00027 | 0.000766 | 0.000281 | 0.001667 | 0.000746178 |
| *Eubacterium_R* | 0.004283 | 0 | 0 | 0 | 0 | 0 |
| *Phascolarctobacterium* | 0.004051 | 0.00384 | 0.002395 | 0.002935 | 0.006446 | 0.003904065 |
| *CAG-110* | 0.003766 | 0 | 0 | 0 | 0 | 0 |
| *Eubacterium_G* | 0.003477 | 0 | 0 | 0 | 0 | 0 |
| *TF01-11* | 0.003054 | 0 | 0 | 0 | 0 | 0 |
| *Sutterella* | 0.003022 | 0.000394 | 0 | 0 | 0.000227 | 0.000155264 |
| *Eubacterium_F* | 0.002877 | 0 | 0 | 0 | 0 | 0 |
| *Enterocloster* | 0.002861 | 0.0001 | 0 | 0 | 0 | 2.50218E-05 |
| *Acutalibacter* | 0.002856 | 0 | 0 | 0 | 0 | 0 |
| *uc_f_Bacteroidaceae* | 0.002845 | 0 | 0 | 0 | 0 | 0 |
| *Catenibacterium* | 0.002748 | 0 | 0 | 0 | 0 | 0 |
| *Butyrivibrio_A* | 0.002696 | 0 | 0 | 0 | 0 | 0 |
| *Eisenbergiella* | 0.002696 | 0 | 0 | 0 | 0 | 0 |
| *RUG714* | 0.002659 | 0 | 0 | 0 | 0 | 0 |
| *UMGS1375* | 0.002633 | 0 | 0 | 0 | 0 | 0 |
| *Ruminococcus_A* | 0.00261 | 0 | 0 | 0 | 0 | 0 |
| *Succinivibrio* | 0.002567 | 0 | 0 | 0.000272 | 0 | 6.79896E-05 |
| *uc_f_Eggerthellaceae* | 0.002565 | 0 | 0 | 0 | 0 | 0 |
| *Clostridium_Q* | 0.002259 | 0.000352 | 0 | 0 | 0 | 8.79555E-05 |
| *Coprococcus_A* | 0.002243 | 0 | 0 | 0 | 0 | 0 |
| *CAG-873* | 0.002215 | 0 | 0 | 0 | 0 | 0 |
| *CAG-41* | 0.002169 | 0 | 0 | 0 | 0 | 0 |
| *UBA7182* | 0.0021 | 0 | 0 | 0 | 0 | 0 |
| *CAG-81* | 0.001949 | 0 | 0 | 0 | 0 | 0 |
| *Oscillibacter* | 0.001946 | 0 | 0 | 0 | 0 | 0 |
| *uc_f_CAG-508* | 0.001889 | 0 | 0 | 0 | 0 | 0 |
| *CAG-45* | 0.001845 | 0 | 0 | 0 | 0 | 0 |
| *CAG-353* | 0.001769 | 0 | 0 | 0 | 0 | 0 |
| *Bilophila* | 0.001721 | 0 | 0 | 0 | 0 | 0 |
| *CHKCI001* | 0.001622 | 0 | 0 | 0 | 0 | 0 |
| *Eubacterium_I* | 0.001427 | 0 | 0 | 0 | 0 | 0 |
| *CAG-462* | 0.001386 | 0.000158 | 0.000485 | 0.000116 | 0.000303 | 0.000265364 |
| *Tidjanibacter* | 0.001372 | 0 | 0 | 0 | 0 | 0 |
| *Parasutterella* | 0.001328 | 0 | 0 | 0.000144 | 0 | 3.59485E-05 |
| *Holdemanella* | 0.001319 | 0 | 0 | 0 | 0 | 0 |
| *Adlercreutzia* | 0.001287 | 0 | 0 | 0 | 0 | 0 |
| *CAG-488* | 0.001262 | 0 | 0 | 0 | 0 | 0 |
| *Anaerotignum* | 0.001227 | 0 | 0 | 0 | 0 | 0 |
| *UBA1191* | 0.001209 | 0 | 0 | 0 | 0 | 0 |
| *Enterococcus_A* | 0 | 0.003649 | 0.00031 | 0.001588 | 0.001237 |  |
| *CAG-1427* | 0.001204 | 0 | 0 | 0 | 0 | 0 |
| *uc_c_Clostridia* | 0.001181 | 0 | 0 | 0 | 0 | 0 |
| *SFTJ01* | 0.001172 | 0 | 0 | 0 | 0 | 0 |
| *CAG-279* | 0.00117 | 0 | 0 | 0 | 0 | 0 |
| *OEMS01* | 0.001128 | 0 | 0 | 0 | 0 | 0 |
| *Ruminococcus_C* | 0.001089 | 0 | 0 | 0 | 0 | 0 |
| *Alistipes_A* | 0.001069 | 0 | 0 | 0 | 0 | 0 |
| *Flavonifractor* | 0.001048 | 0.000989 | 0.000331 | 0.000494 | 0.00057 | 0.00059608 |
| *Mailhella* | 0.001013 | 0 | 0 | 0 | 0 | 0 |
| *uc_o_Oscillospirales* | 0.00097 | 0 | 0 | 0 | 0 | 0 |
| *Ruminiclostridium_E* | 0.000967 | 0 | 0 | 0 | 0 | 0 |
| *Odoribacter* | 0.000938 | 0.000112 | 0.002683 | 0.001691 | 0.003568 | 0.00201356 |
| *PeH17* | 0.000935 | 0 | 0 | 0 | 0 | 0 |
| *uc_f_Acutalibacteraceae* | 0.000933 | 0 | 0 | 0 | 0 | 0 |
| *CAG-603* | 0.000889 | 0 | 0 | 0 | 0 | 0 |
| *51-20* | 0.000887 | 0 | 0 | 0 | 0 | 0 |
| *Zag111* | 0.000885 | 0 | 0 | 0 | 0 | 0 |
| *Romboutsia* | 0.000862 | 0 | 0 | 0 | 0 | 0 |
| *UBA3792* | 0.000859 | 0 | 0 | 0 | 0 | 0 |
| *CAG-127* | 0.000857 | 0 | 0 | 0 | 0 | 0 |
| *Clostridium_N* | 0.000853 | 0 | 0 | 0 | 0 | 0 |
| *Staphylococcus* | 0 | 0.00202 | 0 | 0 | 0 |  |
| *Alloprevotella* | 0 | 0 | 0 | 0 | 0 |  |
| *Blautia* | 0.000827 | 0 | 0 | 0 | 0 | 0 |
| *GCA-900066135* | 0.000825 | 0 | 0 | 0 | 0 | 0 |
| *Clostridium_P* | 0 | 0.001586 | 0.000444 | 0 | 0.000564 |  |
| *Intestinimonas* | 0.000825 | 0 | 0 | 0 | 0.000137 | 3.41828E-05 |
| *Senegalimassilia* | 0.000797 | 0 | 0 | 0 | 0 | 0 |
| *Terrisporobacter* | 0 | 0 | 0 | 0 | 0 |  |
| *Lawsonibacter* | 0.000768 | 0 | 0 | 0 | 0 | 0 |
| *Eubacterium_C* | 0.000745 | 0 | 0 | 0 | 0 | 0 |
| *Enterobacter* | 0 | 0 | 0 | 0.001463 | 0.000567 |  |
| *Clostridium* | 0.000731 | 0.001429 | 0 | 0 | 0 | 0.00035713 |
| *Peptoniphilus_A* | 0 | 0.00124 | 0.001282 | 0.000256 | 0.000987 |  |
| *uc_p_Firmicutes_A* | 0.00071 | 0 | 0 | 0 | 0 | 0 |
| *Ruminococcus* | 0.000699 | 0 | 0 | 0 | 0 | 0 |
| *CAG-495* | 0.000648 | 0 | 0 | 0 | 0 | 0 |
| *UBA9502* | 0.000643 | 0 | 0 | 0 | 0 | 0 |
| *UMGS1071* | 0.000623 | 0 | 0 | 0 | 0 | 0 |
| *Paraprevotella* | 0.00062 | 0 | 0 | 0 | 0 | 0 |
| *Acidaminococcus* | 0.000597 | 0.017085 | 0.000535 | 0.011288 | 0.014967 | 0.010968538 |
| *Intestinibacter* | 0.000586 | 0 | 0 | 0 | 0 | 0 |
| *Veillonella* | 0.000584 | 0.002323 | 0 | 0.000103 | 0.000127 | 0.000638281 |
| *Evtepia* | 0.000581 | 0 | 0 | 0 | 0 | 0 |
| *uc_k_Bacteria* | 0.000552 | 0.001383 | 0.003789 | 0.003439 | 0.002591 | 0.002800526 |
| *Ruthenibacterium* | 0.000549 | 0 | 0 | 0 | 0 | 0 |
| *Citrobacter* | 0 | 0 | 0 | 0.001529 | 0 |  |
| *Longibaculum* | 0.000533 | 0 | 0 | 0 | 0 | 0 |
| *Ruminococcus_B* | 0.000522 | 0 | 0 | 0 | 0 | 0 |
| *Firm-11* | 0.000512 | 0 | 0 | 0 | 0 | 0 |
| *CAG-465* | 0.000508 | 0 | 0 | 0 | 0 | 0 |
| *GCA-900066995* | 0.000473 | 0 | 0 | 0 | 0 | 0 |
| *Eggerthella* | 0.000471 | 0.001498 | 0 | 0.001797 | 0.001037 | 0.001083215 |
| *uc_f_Ruminococcaceae* | 0.000469 | 0 | 0 | 0 | 0 | 0 |
| *UBA2730* | 0.000439 | 0 | 0 | 0 | 0 | 0 |
| *Escherichia* | 0.000434 | 0.08515 | 0.039378 | 0.102719 | 0.069926 | 0.074293385 |
| *uc_f_Anaerovoracaceae* | 0.000414 | 0 | 0 | 0 | 0 | 0 |
| *Butyricicoccus* | 0.000395 | 0 | 0 | 0 | 0 | 0 |
| *Desulfovibrio* | 0.000384 | 0 | 0 | 0 | 0 | 0 |
| *Ligilactobacillus* | 0.000384 | 0 | 0.000209 | 0 | 0 | 5.23686E-05 |
| *RC9* | 0.000381 | 0 | 0 | 0 | 0 | 0 |
| *Coprobacter* | 0.000377 | 0 | 0 | 0.000606 | 0 | 0.000151609 |
| *GCA-900066575* | 0.000363 | 0 | 0 | 0 | 0 | 0 |
| *CAG-238* | 0.000336 | 0 | 0 | 0 | 0 | 0 |
| *CAG-475* | 0 | 0 | 0 | 0 | 0 |  |
| *Longicatena* | 0.000336 | 0 | 0 | 0 | 0.00054 | 0.000135064 |
| *CAG-354* | 0.000333 | 0 | 0 | 0 | 0 | 0 |
| *QALR01* | 0.000315 | 0 | 0 | 0 | 0 | 0 |
| *Phascolarctobacterium_A* | 0.000306 | 0 | 0 | 0 | 0 | 0 |
| *UMGS1441* | 0.000301 | 0 | 0 | 0 | 0 | 0 |
| *CAG-302* | 0.000296 | 0 | 0 | 0 | 0 | 0 |
| *QAMM01* | 0.000287 | 0 | 0 | 0 | 0 | 0 |
| *Clostridium_A* | 0.00028 | 0 | 0 | 0 | 0 | 0 |
| *uc_f_Burkholderiaceae* | 0.000271 | 0 | 0 | 0.000184 | 0 | 4.61079E-05 |
| *CAG-196* | 0.000269 | 0 | 0 | 0 | 0 | 0 |
| *Hungatella_A* | 0.000262 | 0 | 0 | 0 | 0 | 0 |
| *Peptostreptococcus* | 0 | 0.000664 | 0 | 0 | 0 |  |
| *Hungatella* | 0 | 0.000334 | 0 | 0.000228 | 0 |  |
| *Negativibacillus* | 0.000257 | 0 | 0 | 0 | 0 | 0 |
| *UMGS1781* | 0.000234 | 0 | 0 | 0 | 0 | 0 |
| *UBA1820* | 0.00023 | 0 | 0 | 0 | 0 | 0 |
| *Schaedlerella* | 0.000223 | 0 | 0 | 0 | 0 | 0 |
| *Brachyspira* | 0.000223 | 0 | 0 | 0 | 0 | 0 |
| *CAG-484* | 0 | 0 | 0 | 0 | 0 |  |
| *Kineothrix* | 0 | 0 | 0 | 0 | 0 |  |
| *CAG-314* | 0 | 0 | 0 | 0 | 0 |  |
| *uc_f_Enterobacteriaceae* | 0 | 0 | 0 | 0.000569 | 0 |  |
| *OEMR01* | 0.000214 | 0 | 0 | 0 | 0 | 0 |
| *CAG-307* | 0.000207 | 0 | 0 | 0 | 0 | 0 |
| *Faecalicoccus* | 0 | 0 | 0 | 0 | 0 |  |
| *uc_f_Muribaculaceae* | 0.000205 | 0 | 0 | 0 | 0 | 0 |
| *Turicibacter* | 0.000205 | 0.000376 | 0.000688 | 0 | 0.000303 | 0.000341847 |
| *Butyricimonas* | 0.000205 | 0 | 0 | 0 | 0 | 0 |
| *CAG-180* | 0 | 0 | 0 | 0 | 0 |  |
| *Megasphaera* | 0.000198 | 0 | 0 | 0 | 0 | 0 |
| *Slackia_A* | 0.000188 | 0 | 0 | 0 | 0 | 0 |
| *Marvinbryantia* | 0.000179 | 0 | 0 | 0 | 0 | 0 |
| *UBA2883* | 0.000175 | 0 | 0 | 0 | 0 | 0 |
| *uc_o_Peptostreptococcales* | 0 | 0 | 0 | 0 | 0 |  |
| *UBA7057* | 0.000172 | 0 | 0 | 0 | 0 | 0 |
| *OF09-33XD* | 0.00017 | 0 | 0 | 0 | 0 | 0 |
| *Lactobacillus* | 0 | 0.000185 | 0.000147 | 0 | 0.00012 |  |
| *Oxalobacter* | 0.000154 | 0 | 0 | 0 | 0 | 0 |
| *Succiniclasticum* | 0.000145 | 0 | 0 | 0 | 0 | 0 |
| *Acetitomaculum* | 0 | 0 | 0 | 0 | 0 |  |
| *uc_f_Oscillospiraceae* | 0.000142 | 0 | 0 | 0 | 0 | 0 |
| *G11* | 0 | 0 | 0 | 0 | 0 |  |
| *Frisingicoccus* | 0.000142 | 0 | 0 | 0 | 0 | 0 |
| *uc_f_Erysipelatoclostridiaceae* | 0 | 0 | 0 | 0 | 0 |  |
| *UBA6985* | 0.000136 | 0 | 0 | 0 | 0 | 0 |
| *CAG-115* | 0.000126 | 0 | 0 | 0 | 0 | 0 |
| *Emergencia* | 0.000126 | 0 | 0 | 0 | 0 | 0 |
| *Olsenella_E* | 0.000124 | 0 | 0 | 0 | 0 | 0 |
| *Mediterraneibacter_A* | 0 | 0 | 0 | 0 | 0 |  |
| *Massiliomicrobiota* | 0.000115 | 0 | 0 | 0 | 0 | 0 |
| *AM51-8* | 0 | 0 | 0 | 0 | 0 |  |
| *Mesosutterella* | 0 | 0 | 0 | 0 | 0 |  |
| *Allisonella* | 0 | 0 | 0 | 0 | 0 |  |
| *Finegoldia* | 0 | 0.000124 | 0 | 0 | 0 |  |
| *Anaerotruncus* | 0 | 0 | 0 | 0 | 0 |  |
| *Monoglobus* | 0.00011 | 0 | 0 | 0 | 0 | 0 |
| *CAG-313* | 0 | 0 | 0 | 0 | 0 |  |
| *UBA9475* | 0 | 0 | 0 | 0 | 0 |  |
| *Sporobacter* | 0.000106 | 0 | 0 | 0 | 0 | 0 |
| *Anaerococcus* | 0 | 0 | 0 | 0 | 0 |  |
| *Ezakiella* | 0 | 0 | 0 | 0 | 0 |  |
| *CAG-628* | 0 | 0 | 0 | 0 | 0 |  |
| *uc_o_Lachnospirales* | 0 | 0 | 0 | 0 | 0 |  |
| *Methanosphaera* | 0.000106 | 0 | 0 | 0 | 0 | 0 |
| *Parolsenella* | 0.000103 | 0 | 0 | 0 | 0 | 0 |
| *uc_f_Acetivibrionaceae* | 0 | 0 | 0 | 0 | 0 |  |
| *UBA733* | 0 | 0 | 0 | 0 | 0 |  |
| *Helicobacter_C* | 0 | 0 | 0 | 0 | 0 |  |
| *Fenollaria* | 0 | 0 | 0.000163 | 0 | 0 |  |
| *UBA6382* | 0 | 0 | 0 | 0 | 0 |  |
| *Fournierella* | 0 | 0 | 0 | 0 | 0 |  |
| *UMGS416* | 0 | 0 | 0 | 0 | 0 |  |
| *CAG-95* | 0 | 0 | 0 | 0 | 0 |  |
| *Peptoniphilus_C* | 0 | 0 | 0 | 0 | 0 |  |
| *SFVR01* | 0 | 0 | 0 | 0 | 0 |  |
| *Ruminococcus_F* | 0 | 0 | 0 | 0 | 0 |  |
| *Anaeromassilibacillus* | 0 | 0 | 0 | 0 | 0 |  |
| *uc_f_CAG-822* | 0.000101 | 0 | 0 | 0 | 0 | 0 |
| *uc_f_Gastranaerophilaceae* | 0 | 0 | 0 | 0 | 0 |  |
| *CAG-878* | 0 | 0 | 0 | 0 | 0 |  |
| *Ruminiclostridium* | 0 | 0 | 0 | 0 | 0 |  |
| *Mitsuokella* | 0 | 0 | 0 | 0.000109 | 0 |  |
| *UBA9414* | 0 | 0 | 0 | 0 | 0 |  |
| *Eubacterium* | 0 | 0 | 0 | 0.0001 | 0 |  |
| *Enteroscipio* | 0 | 0 | 0 | 0 | 0 |  |
| *GCA-900066905* | 0 | 0 | 0 | 0 | 0 |  |
| *Porphyromonas_A* | 0 | 0 | 0 | 0 | 0 |  |
| *Anaerofustis* | 0 | 0 | 0 | 0 | 0 |  |
| *Citrobacter_A* | 0 | 0 | 0 | 0 | 0 |  |
| *uc_o_RF39* | 0 | 0 | 0 | 0 | 0 |  |
| *Achromobacter* | 0 | 0 | 0 | 0 | 0 |  |
| *Duncaniella* | 0 | 0 | 0 | 0 | 0 |  |
