# Supplementary material for: Effect of cryopreservation medium conditions on growth and isolation of gut anaerobes from human faecal samples
Source: Microbiome. 2022 May 30;10:80. doi: 10.1186/s40168-022-01267-2 (PMC9150342; doi:10.1186/s40168-022-01267-2)
Supplement: Supplementary file 10 — Additional file 9: Supplementary Table S6: Associations between richness in the cultured fractions and the original richness, the preservation conditions and dilutions (n=129). [file 40168_2022_1267_MOESM10_ESM.docx]

| **Supplementary Table S6: Associations between richness in the cultured fractions and the original richness, the preservation conditions and dilutions (n=129).** | | | | |
| --- | --- | --- | --- | --- |
| **Variable** | **Estimate** | **glm.std.coef** | **glm.pvalue** | **glm.FDR** |
| Dilution | 0.0215882 | 0.0041725 | 9.03E-07 | 1.30E-06 |
| PreservationP2 | 0.0144943 | 0.0109411 | 0.18771 |  |
| PreservationP3 | 0.0146594 | 0.0110342 | 0.18646 |  |
| PreservationP4 | -0.0043553 | 0.0102989 | 0.67311 |  |
| RichnessF | -0.0008683 | 0.0002852 | 0.00285 | 0.003483 |
