# Supplementary material for: Effect of cryopreservation medium conditions on growth and isolation of gut anaerobes from human faecal samples
Source: Microbiome. 2022 May 30;10:80. doi: 10.1186/s40168-022-01267-2 (PMC9150342; doi:10.1186/s40168-022-01267-2)
Supplement: Supplementary file 11 — Additional file 10: Supplementary Table S7: Summary of the number of isolates and the number of recovered sequences from 16S rDNA sequencing of the isolates per individual and preservation condition. [file 40168_2022_1267_MOESM11_ESM.docx]

| **Supplementary Table S7: Summary of the number of isolates and the number of recovered sequences from 16S rDNA sequencing of the isolates per individual and preservation condition.** | | | | |
| --- | --- | --- | --- | --- |
| **Individual** | **Preservation** | **Isolates** | **Sequenced** | **%** |
| SC03 | P1 | 94 | 82 | 0.87 |
| SC03 | P2 | 94 | 91 | 0.97 |
| SC03 | P3 | 94 | 85 | 0.90 |
| SC03 | P4 | 94 | 50 | 0.53 |
| SC08 | P1 | 94 | 57 | 0.61 |
| SC08 | P2 | 94 | 81 | 0.86 |
| SC08 | P3 | 94 | 69 | 0.73 |
| SC08 | P4 | 94 | 86 | 0.91 |
| SC17 | P1 | 94 | 39 | 0.41 |
| SC17 | P2 | 94 | 74 | 0.79 |
| SC17 | P3 | 72 | 69 | 0.73 |
| SC17 | P4 | 94 | 80 | 0.85 |
| SC18 | P1 | 94 | 84 | 0.89 |
| SC18 | P2 | 94 | 80 | 0.85 |
| SC18 | P3 | 94 | 86 | 0.91 |
| SC18 | P4 | 94 | 87 | 0.93 |
| SC41 | P1 | 94 | 86 | 0.91 |
| SC41 | P2 | 94 | 91 | 0.97 |
| SC41 | P3 | 94 | 92 | 0.98 |
| SC41 | P4 | 94 | 63 | 0.67 |
| SC21 | P1 | 94 | 87 | 0.93 |
| SC21 | P2 | 94 | 90 | 0.96 |
| SC21 | P3 | 94 | 88 | 0.94 |
| SC21 | P4 | 94 | 77 | 0.82 |
| SC22 | P1 | 94 | 80 | 0.85 |
| SC22 | P2 | 94 | 65 | 0.69 |
| SC22 | P3 | 89 | 89 | 0.95 |
| SC22 | P4 | 94 | 92 | 0.98 |
| SC23 | P1 | 94 | 63 | 0.67 |
| SC23 | P2 | 94 | 82 | 0.87 |
| SC23 | P3 | 94 | 94 | 1.00 |
| SC23 | P4 | 83 | 71 | 0.76 |
| SC29 | P1 | 94 | 77 | 0.82 |
| SC29 | P2 | 94 | 89 | 0.95 |
| SC29 | P3 | 94 | 69 | 0.73 |
| SC29 | P4 | 94 | 84 | 0.89 |
| SC36 | P1 | 94 | 45 | 0.48 |
| SC36 | P2 | 94 | 84 | 0.89 |
| SC36 | P3 | 94 | 85 | 0.90 |
| SC36 | P4 | 94 | 92 | 0.98 |
| SC42 | P1 | 94 | 48 | 0.51 |
| SC42 | P2 | 94 | 75 | 0.80 |
| SC42 | P3 | 84 | 70 | 0.74 |
| SC42 | P4 | 94 | 67 | 0.71 |
|  |  |  | Median | 0.87 |
|  |  |  | Mean | 0.82 |
|  |  |  | Range | [41 - 100] |
|  |  |  |  |  |
|  | Isolates | 4088 | 3395 |  |
|  |  |  |  |  |
