# Supplementary material for: Effect of cryopreservation medium conditions on growth and isolation of gut anaerobes from human faecal samples
Source: Microbiome. 2022 May 30;10:80. doi: 10.1186/s40168-022-01267-2 (PMC9150342; doi:10.1186/s40168-022-01267-2)
Supplement: Supplementary file 12 — Additional file 11: Supplementary Table S8: Proportion of taxa per total sequences for each preservation condition at genus and species level. [file 40168_2022_1267_MOESM12_ESM.docx]

| **Supplementary Table S8: Proportion of taxa per total sequences for each preservation condition at genus and species level.** | | | | | | | |
| --- | --- | --- | --- | --- | --- | --- | --- |
| **Preservation condition** | **Species** | **Genera** | **Total sequenced** | **Proportion of genera per total sequences** | **Proportion of species per total sequences** | **Proportion of genera in P1 compared to Px** | **Proportion of species in P1 compared to Px2** |
| P1 | 72 | 36 | 686 | 5% | 10% |  |  |
| P2 | 62 | 30 | 804 | 4% | 8% | 1.41 | 1.36 |
| P3 | 59 | 29 | 858 | 3% | 7% | 1.55 | 1.53 |
| P4 | 66 | 33 | 774 | 4% | 9% | 1.23 | 1.23 |
