# Supplementary material for: Effect of cryopreservation medium conditions on growth and isolation of gut anaerobes from human faecal samples
Source: Microbiome. 2022 May 30;10:80. doi: 10.1186/s40168-022-01267-2 (PMC9150342; doi:10.1186/s40168-022-01267-2)
Supplement: Supplementary file 13 — Additional file 12: Supplementary Table S9: Presence/absence of genera between culture-independent (feacal samples), cultured fractions and isolates, detection threshold >0.01%. [file 40168_2022_1267_MOESM13_ESM.docx]

| **Supplementary Table S9: Presence/absence of genera between culture-independent (feacal samples), cultured fractions and isolates, detection threshold >0.01%.** | | | |
| --- | --- | --- | --- |
| **Genera** | **Isolated** | **Cultured fraction** | **Faecal samples** |
| *Acetatifactor* |  |  | 1 |
| *Acidaminococcus* |  | 1 |  |
| *Acutalibacter* |  |  | 1 |
| *Adlercreutzia* | 1 |  | 1 |
| *Agathobacter* |  |  | 1 |
| *Agathobaculum* |  |  | 1 |
| *Akkermansia* | 1 |  | 1 |
| *Alistipes* | 1 |  | 1 |
| *Alistipes_A* | 1 |  | 1 |
| *Anaerobutyricum* |  |  | 1 |
| *Anaerostipes* |  |  | 1 |
| *Anaerotignum* |  |  | 1 |
| *Bacteroides* | 1 | 1 | 1 |
| *Bariatricus* |  |  | 1 |
| *Barnesiella* | 1 |  |  |
| *Bifidobacterium* | 1 | 1 | 1 |
| *Bilophila* | 1 |  | 1 |
| *Blautia_A* |  |  | 1 |
| *Brachyspira* |  |  | 1 |
| *Butyricicoccus* |  |  | 1 |
| *Butyricimonas* | 1 |  |  |
| *Butyrivibrio_A* |  |  | 1 |
| *CAG-462* | 1 | 1 | 1 |
| *CAG-831* |  | 1 | 1 |
| *Citrobacter* |  | 1 |  |
| *Clostridium* | 1 | 1 |  |
| *Clostridium_A* | 1 |  |  |
| *Clostridium_N* |  |  | 1 |
| *Clostridium_P* |  | 1 |  |
| *Clostridium_Q* |  | 1 | 1 |
| *Collinsella* | 1 | 1 | 1 |
| *Coprobacillus* | 1 |  |  |
| *Coprobacter* | 1 | 1 |  |
| *Coprococcus* |  |  | 1 |
| *Coprococcus_A* |  |  | 1 |
| *Cutibacterium* | 1 |  |  |
| *Desulfovibrio* | 1 |  |  |
| *Dialister* |  | 1 | 1 |
| *Dorea* |  | 1 | 1 |
| *Eggerthella* | 1 | 1 | 1 |
| *Eisenbergiella* | 1 |  | 1 |
| *Enorma* | 1 |  |  |
| *Enterobacter* |  | 1 |  |
| *Enterocloster* | 1 | 1 | 1 |
| *Enterococcus_A* | 1 | 1 |  |
| *Enterococcus_B* | 1 | 1 |  |
| *Enteroscipio* | 1 |  |  |
| *Erysipelatoclostridium* |  | 1 | 1 |
| *Escherichia* | 1 | 1 | 1 |
| *Eubacterium* | 1 | 1 |  |
| *Eubacterium_C* |  |  | 1 |
| *Eubacterium_F* |  |  | 1 |
| *Eubacterium_G* |  |  | 1 |
| *Eubacterium_I* |  |  | 1 |
| *Eubacterium_R* |  |  | 1 |
| *Evtepia* |  |  | 1 |
| *Faecalibacterium* |  |  | 1 |
| *Fenollaria* |  | 1 |  |
| *Finegoldia* |  | 1 |  |
| *Flavonifractor* | 1 | 1 | 1 |
| *Fusicatenibacter* |  |  | 1 |
| *Gemmiger* |  |  | 1 |
| *Gordonibacter* | 1 |  |  |
| *Hafnia* | 1 |  |  |
| *Holdemanella* | 1 |  | 1 |
| *Hungatella* | 1 | 1 |  |
| *Intestinibacter* |  |  | 1 |
| *Intestinimonas* | 1 | 1 | 1 |
| *Lachnospira* |  |  | 1 |
| *Lactobacillus* |  | 1 |  |
| *Lactococcus* | 1 |  |  |
| *Lapidilactobacillus* | 1 |  |  |
| *Lawsonibacter* |  |  | 1 |
| *Ligilactobacillus* | 1 | 1 |  |
| *Longicatena* | 1 | 1 |  |
| *Marseille-P4683* | 1 |  |  |
| *Mediterraneibacter* |  | 1 | 1 |
| *Methanobrevibacter_A* |  |  | 1 |
| *Mitsuokella* |  | 1 |  |
| *Odoribacter* | 1 | 1 | 1 |
| *Parabacteroides* | 1 | 1 | 1 |
| *Paraprevotella* |  |  | 1 |
| *Parasutterella* |  | 1 | 1 |
| *Parvimonas* | 1 |  |  |
| *Pauljensenia* | 1 |  |  |
| *Peptoniphilus_A* | 1 | 1 |  |
| *Peptostreptococcus* |  | 1 |  |
| *Phascolarctobacterium* | 1 | 1 | 1 |
| *Phocaeicola* | 1 | 1 | 1 |
| *Prevotella* | 1 |  | 1 |
| *Prevotellamassilia* |  | 1 | 1 |
| *Pseudomonas_E* | 1 |  |  |
| *Raoultibacter* | 1 |  |  |
| *Roseburia* |  |  | 1 |
| *Ruminiclostridium_E* |  |  | 1 |
| *Ruminococcus_A* |  |  | 1 |
| *Ruminococcus_B* |  |  | 1 |
| *Ruminococcus_C* |  |  | 1 |
| *Ruminococcus_D* |  |  | 1 |
| *Ruminococcus_E* | 1 |  | 1 |
| *Ruthenibacterium* | 1 |  | 1 |
| *Senegalimassilia* |  |  | 1 |
| *Staphylococcus* | 1 | 1 |  |
| *Streptococcus* | 1 | 1 | 1 |
| *Succinivibrio* |  | 1 | 1 |
| *Sutterella* |  | 1 | 1 |
| *Tidjanibacter* | 1 |  |  |
| *Turicibacter* |  | 1 |  |
| *Varibaculum* | 1 |  |  |
| *Veillonella* | 1 | 1 |  |
| *Vibrio* | 1 |  |  |
